# Anti-Inflammatory and Anti-Migratory Activities of Isoquinoline-1-Carboxamide Derivatives in LPS-Treated BV2 Microglial Cells via Inhibition of MAPKs/NF-κB Pathway

**DOI:** 10.3390/ijms21072319

**Published:** 2020-03-27

**Authors:** Ha Thi Thu Do, Bich Phuong Bui, Seongrak Sim, Jae-Kyung Jung, Heesoon Lee, Jungsook Cho

**Affiliations:** 1 College of Pharmacy, Dongguk University-Seoul, Goyang, Gyeonggi 10326, Korea; doha201191@gmail.com (H.T.T.D.); bichphuong2306@gmail.com (B.P.B.); 2 College of Pharmacy, Chungbuk National University, Osong, Cheongju 28160, Korea; stephanoes@chungbuk.ac.kr (S.S.); orgjkjung@chungbuk.ac.kr (J.-K.J.)

**Keywords:** isoquinoline-1-carboxamide, nuclear factor-kappa B (NF-κB), mitogen-activated protein kinases (MAPKs), BV2 microglial cells, intracellular signaling, neuroinflammation, cell migration, neurodegeneration

## Abstract

Eleven novel isoquinoline-1-carboxamides (HSR1101~1111) were synthesized and evaluated for their effects on lipopolysaccharide (LPS)-induced production of pro-inflammatory mediators and cell migration in BV2 microglial cells. Three compounds (HSR1101~1103) exhibited the most potent suppression of LPS-induced pro-inflammatory mediators, including interleukin (IL)-6, tumor necrosis factor-alpha, and nitric oxide (NO), without significant cytotoxicity. Among them, only *N*-(2-hydroxyphenyl) isoquinoline-1-carboxamide (HSR1101) was found to reverse LPS-suppressed anti-inflammatory cytokine IL-10, so it was selected for further characterization. HSR1101 attenuated LPS-induced expression of inducible NO synthase and cyclooxygenase-2. Particularly, HSR1101 abated LPS-induced nuclear translocation of NF-κB through inhibition of IκB phosphorylation. Furthermore, HSR1101 inhibited LPS-induced cell migration and phosphorylation of mitogen-activated protein kinases (MAPKs) including extracellular signal-regulated kinase 1/2, c-Jun N-terminal kinase, and p38 MAPK. The specific MAPK inhibitors, U0126, SP600125, and SB203580, suppressed LPS-stimulated pro-inflammatory mediators, cell migration, and NF-κB nuclear translocation, indicating that MAPKs may be the upstream kinase of NF-κB signaling. Collectively, these results demonstrate that HSR1101 is a potent and promising compound suppressing LPS-induced inflammation and cell migration in BV2 microglial cells, and that inhibition of the MAPKs/NF-κB pathway mediates its anti-inflammatory and anti-migratory effects. Based on our findings, HSR1101 may have beneficial impacts on various neurodegenerative disorders associated with neuroinflammation and microglial activation.

## 1. Introduction

Neurodegeneration is described as the slow and progressive dysfunction and loss of neurons or axons in the central nervous system (CNS). It is the primary pathological feature of acute and chronic disorders such as Parkinson’s disease (PD), Alzheimer’s disease (AD), and multiple sclerosis [1]. Growing evidence indicates that immune responses, including neuroinflammation, are directly implicated in the processes of neurodegeneration [1]. Particularly, microglial cells, which are the resident innate immune cells in the CNS, play critical roles in chronic immune activation and neuroinflammation [2,3]. On the one hand, activated microglial cells scavenge dead cells from the CNS and release either several neurotrophic factors that regulate neuronal survival or anti-inflammatory cytokines, including interleukin (IL)-4 and IL-10, to combat neurodegenerative conditions [4,5,6]. On the other hand, over-activation of microglial cells causes copious immune responses and inflammation, eventually leading to various types of neurodegenerative diseases [1,7,8]. The over-stimulated microglial cells have been demonstrated to induce detrimental neuronal damage as well as neurodegenerative processes through excess production of multiple pro-inflammatory mediators, such as IL-6, tumor necrosis factor (TNF)-α, and nitric oxide (NO). In addition, inducible NO synthase (iNOS), cyclooxygenase (COX)-2, and other enzymes associated with the inflammatory processes may also be induced [9,10]. In response to injury or inflammatory stimuli, microglial cells are rapidly activated to participate in pathological responses, such as migration to the affected sites and the release of various inflammatory molecules [11]. Therefore, increased migration may also be considered as a feature of activated microglial cells [11,12].

The role of microglia-mediated neuroinflammation in the progressive nature of many neurodegenerative disorders has been demonstrated in models of microglial cells activated by pro-inflammatory stimuli [2]. Lipopolysaccharide (LPS), an endotoxin expressed in the external membrane of Gram-negative bacteria, has been widely employed to provoke the neuroinflammatory processes through the secretion of various cytokines and eicosanoids [2,13]. It has been reported that LPS-activated microglia can initiate neuronal cell damage [14,15], resulting in delayed and progressive degeneration of neurons over time [14,16]. The present study employed LPS-treated BV2 cells, a murine microglial cell line, as a model to evaluate the effects of synthesized isoquinoline-1-carboxamide derivatives on the production of pro-inflammatory mediators and cell migration [17,18].

It is well-recognized that activation of the nuclear factor-kappa B (NF-κB) family plays a critical role in inflammation. The nuclear translocation of NF-κB for stimulation of gene transcription is regulated by the phosphorylation of proteins called inhibitors of kappa B (IκBs), especially IκBα [19]. The activation of microglial cells by LPS is associated with the NF-κB signaling pathway [9,10,20]. In addition, it has been revealed that the transcription and production of pro-inflammatory factors in activated microglial cells involve the mitogen-activated protein kinases (MAPKs), including extracellular signal-regulated kinase 1/2 (ERK1/2), c-Jun N-terminal kinase (JNK) and p38 MAPK [20,21,22,23]. Thus, inhibiting NF-κB and MAPK pathways may be promising approaches to combat the progression of neurodegenerative processes associated with inflammation and microglial activation.

**Figure 1:**
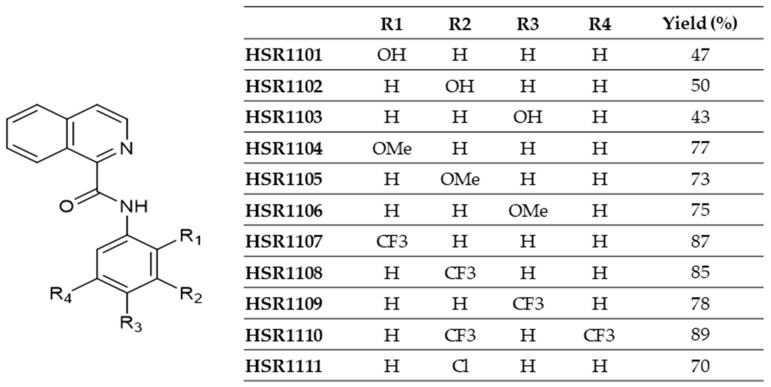
Chemical structures and yield of isoquinoline-1-carboxamide derivatives.

Recently, we reported 1,2,3,4-tetrahydroquinoline derivative (ELC-D-2) as a potential candidate for the treatment of neurodegenerative disorders through inhibition of pro-inflammatory mediators and cell migration [20]. In an effort to discover novel anti-inflammatory agents, we changed tetrahydroquinoline to isoquinoline scaffold to generate a series of isoquinoline-1-carboxamide derivatives with *N*-hydroxyphenyl, *N*-methoxyphenyl, *N*-(trifluoromethyl)phenyl, *N*-bis(trifluoromethyl)phenyl or *N*-chlorophenyl substituents (Figure 1). The present study aimed to evaluate the effects of the newly synthesized isoquinoline-1-carboxamides on LPS-stimulated inflammation and cell migration in BV2 microglial cells. In addition, we investigated the signaling pathways involved in their actions. We found that, among the eleven tested compounds, *N*-(2-hydroxyphenyl)isoquinoline-1-carboxamide (HSR1101) potently inhibited LPS-induced production of the pro-inflammatory mediators including IL-6, TNF-α, and NO in BV2 cells. It also inhibited LPS-induced BV2 cell migration. Moreover, HSR1101 significantly reversed the level of IL-10, an anti-inflammatory cytokine, suppressed by LPS treatment. Finally, our findings indicated that inhibition of MAPKs and NF-κB pathways by HSR1101 mediates its anti-inflammatory and anti-migratory effects in BV2 cells.

## 2. Results

### 2.1. Effects of Isoquinoline-1-carboxamides on Viabilities of BV2 Cells

**Figure 2:**
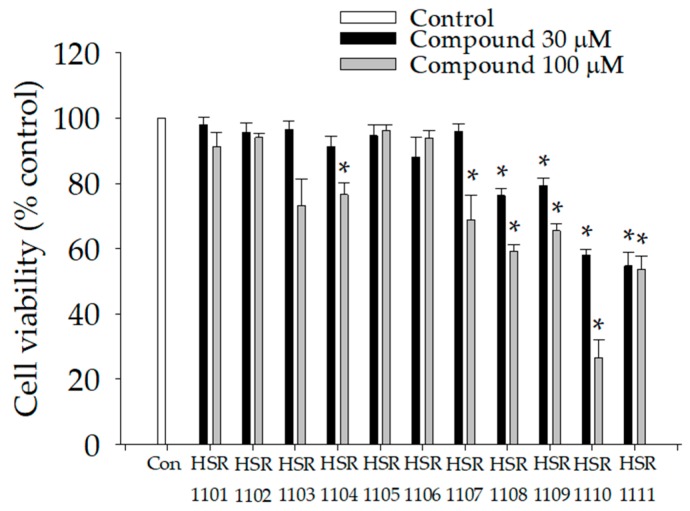
Effects of isoquinoline-1-carboxamides on the viabilities of BV2 cells. BV2 cells were treated with a series of concentrations of these compounds for 24 h. Then, MTT assays were performed, as described in the Materials and methods section. Data are expressed as mean ± SEM of at least three independent experiments. * *p* < 0.05 vs the vehicle-treated control. Con, control.

Prior to the evaluation of their anti-inflammatory and anti-migratory activities, we first examined the effects of isoquinoline-1-carboxamide derivatives on BV2 cell viabilities. The cells were treated with the tested compounds at the concentrations of 30 or 100 µM for 24 h, and then MTT assays were performed. We found that the derivatives with *N*-hydroxyphenyl (HSR1101~1103) and *N*-methoxyphenyl (HSR1105 and 1106) substituents revealed no significant cytotoxicity at the concentrations examined in this study (Figure 2). In contrast, the derivatives with *N*-(trifluoromethyl)phenyl (HSR1108 and 1109), *N*-bis(trifluoromethyl)phenyl (HSR1110) or *N*-chlorophenyl (HSR1111) substituents showed prominent cytotoxicity at both 30 and 100 µM concentrations. Although the derivatives with *N*-methoxyphenyl (HSR1104) and *N*-(trifluoromethyl)phenyl (HSR1107) substituents did not exhibit any noticeable cytotoxicity at 30 µM, considerable cytotoxicity was observed at the concentration of 100 µM. Based on these findings, five compounds (HSR1101~1103, 1105, and 1106) without showing significant cytotoxicity up to 100 µM were selected for further investigations.

### 2.2. Effects of Isoquinoline-1-carboxamides on the Production of Pro- and Anti-Inflammatory Mediators in LPS-Treated BV2 Cells

**Figure 3:**
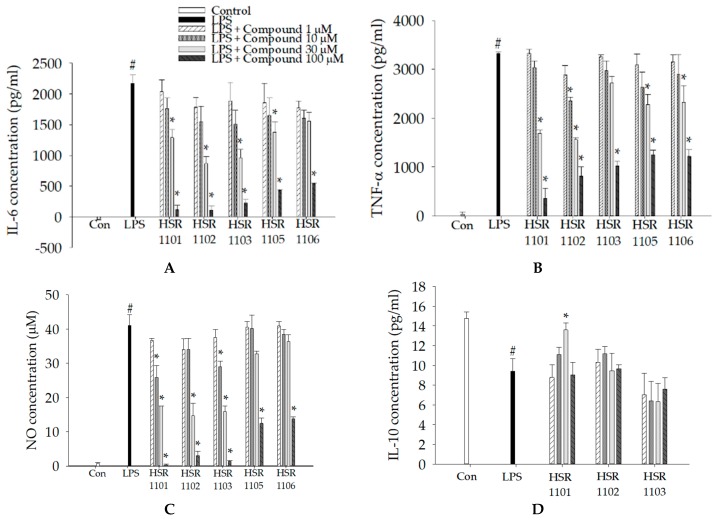
Effects of HSR1101~1103, 1105, and 1106 on pro- and anti-inflammatory mediators in lipopolysaccharide (LPS)-treated BV2 cells. BV2 cells were co-treated with 1 μg/mL LPS and a series of concentrations of tested compounds for 24 h. Culture media were then analyzed for interleukin (IL)-6 (**A**), TNF-α (**B**), NO (**C**), and IL-10 (**D**) levels, as described in the Materials and methods section. Data are expressed as mean ± SEM of at least three independent experiments. # *p* < 0.05 and * *p* < 0.05 vs the vehicle-treated control and LPS-treated groups, respectively. Con, control; LPS, lipopolysaccharide; IL, interleukin; TNF-α, tumor necrosis factor-alpha; NO, nitric oxide.

We next examined the effects of the five selected derivatives (HSR1101~1103, 1105, and 1106) on the production of LPS-stimulated pro-inflammatory mediators. It was found that LPS treatment markedly enhanced the production of the pro-inflammatory factors IL-6, TNF-α, and NO (Figure 3). Meanwhile, simultaneous treatment with LPS and the testing compounds reduced the production of these mediators in concentration-dependent manners. The calculated IC_50_ values of these compounds are indicated in Table 1. The derivatives with the *N*-hydroxyphenyl substituents (HSR1101~1103) exhibited potent and comparable inhibitory effects on the LPS-induced IL-6 and NO with IC_50_ values in the range of 20–40 µM (Figure 3A,C, and Table 1). In contrast, two derivatives with *N*-methoxyphenyl substituents (HSR1105 and 1106) showed less potent inhibition of IL-6 and NO, with IC_50_ values in the range of 40–80 µM (Figure 3A,C, and Table 1). Whereas HSR1101 and 1102 possessed the more potent suppression of TNF-α with IC_50_ values less than 30 µM, the remaining compounds HSR1103, 1105, and 1106 showed only mild inhibition with IC_50_ values of approximately 70 µM (Figure 3B and Table 1). Among these five tested compounds, the derivatives with the *N*-hydroxyphenyl substituent (HSR1101~1103) exhibited more powerful inhibitions for all three pro-inflammatory mediators. Therefore, these three compounds were selected to evaluate whether they show any impact on the production of anti-inflammatory cytokine IL-10.

Results showed that IL-10 production was significantly reduced in the LPS-treated BV2 cells, as compared with the vehicle-treated control cells (Figure 3D). Among the three compounds tested, only HSR1101 (*N*-(2-hydroxyphenyl)isoquinoline-1-carboxamide) revealed a tendency to reverse the LPS-suppressed IL-10 level, with the statistical significance at the concentration of 30 μM (Figure 3D). In contrast, there are no significant alterations of IL-10 levels exhibited by HSR1102 or 1103. Collectively, HSR1101 is the only compound not only inhibiting pro-inflammatory mediators such as IL-6, TNF-α, and NO, but also increasing anti-inflammatory cytokine IL-10 in the LPS-treated BV2 cells. Consequently, HSR1101 was selected for further characterization in this study.

In addition, we further examined the effects of post-treated HSR1101 on the secretion of pro- and anti-inflammatory mediators in LPS-activated BV2 cells. After pre-treatment with LPS for 30 min, the cells were treated with HSR1101 at the final concentrations of 30 and 100 μM for 24 h. In consistent with our findings obtained from simultaneous treatments with LPS and HSR1101 (Figure 3), we observed that the post-treated HSR1101 also dramatically inhibited the production of pro-inflammatory mediators including IL-6, TNF-α, and NO at both concentrations tested (data not shown). We also observed that the post-treated HSR1101 significantly enhanced the production of anti-inflammatory IL-10 at 30 μM in LPS-treated BV2 cells (data not shown).

### 2.3. Effect of HSR1101 on LPS-Stimulated iNOS and COX-2 Expression in BV2 Cells

**Figure 4:**
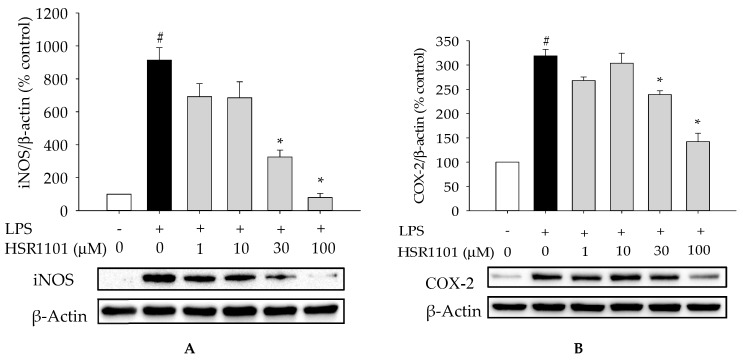
HSR1101 inhibited LPS-induced expression of inducible NO synthase (iNOS) and COX-2 in BV2 cells. BV2 cells were co-treated with 1 μg/mL LPS and a series of concentrations of HSR1101 for 24 h. Western blotting analyses were conducted using anti-iNOS (**A**) and anti-COX-2 (**B**) antibodies, as described in the Materials and methods section. β-Actin was used for normalizing iNOS and COX-2. Representative blots are displayed. Data are expressed as mean ± SEM of at least three independent experiments. # *p* < 0.05 and * *p* < 0.05 vs the vehicle-treated control and LPS-treated groups, respectively. LPS, lipopolysaccharide; iNOS, inducible nitric oxide synthase; COX-2, cyclooxygenase-2.

To evaluate the effect of HSR1101 on iNOS and COX-2 expression, Western blotting analysis was performed. Our results demonstrated that LPS treatment dramatically augmented the expression levels of iNOS and COX-2 in BV2 cells. Simultaneous treatment with LPS and HSR1101 at 30 or 100 µM dramatically decreased the LPS-induced iNOS and COX-2 expression (Figure 4). These results suggest that the inhibition of iNOS and COX-2 expression by HSR1101 may contribute to its anti-inflammatory effect in LPS-stimulated BV2 cells.

Along with HSR1101, we also explored the effects of HSR1102 and 1103 on the expression of iNOS and COX-2 in LPS-treated BV2 cells. As expected, both compounds also inhibited LPS-induced iNOS and COX-2 expression, with comparable to or less efficacy than HSR1101 at 30 and 100 µM (data not shown).

### 2.4. Effects of HSR1101 on LPS-Induced NF-κB Translocation and IκBα Phosphorylation in BV2 Cells

**Figure 5:**
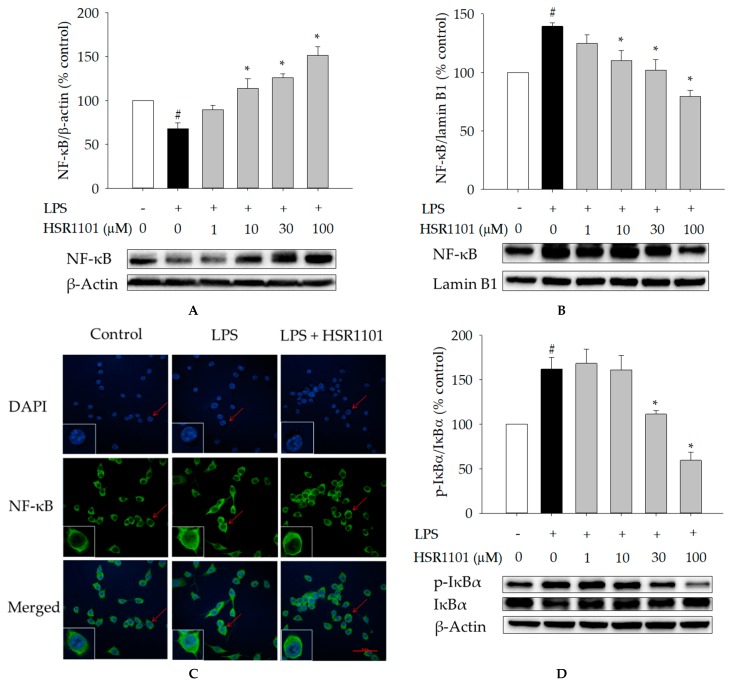
HSR1101 inhibited LPS-induced nuclear translocation of NF-κB through suppression of IκBα phosphorylation in BV2 cells. BV2 cells were co-treated with 1 μg/mL LPS and a series of concentrations of HSR1101 for 24 h. Western blotting analyses for cytosolic (**A**) and nuclear (**B**) extracts were conducted using anti-NF-κB p65 subunit antibody. β-Actin and lamin B1 were used for normalizing cytosolic and nuclear NF-κB, respectively. Immunofluorescence images show inhibition of NF-κB translocation by HSR1101 (**C**). The red arrows indicate the magnified cells shown in each image. Scale bar, 50 μm. Western blotting analyses were conducted using anti-phospho-IκBα and anti-IκBα antibodies (**D**). β-Actin was used for normalizing phosphor-IκBα. Representative blots are displayed. Data are expressed as mean ± SEM of at least three independent experiments. # *p* < 0.05 and * *p* < 0.05 vs the vehicle-treated control and LPS-treated groups, respectively. LPS, lipopolysaccharide; NF-κB, nuclear factor-kappa B; IκBα, inhibitor of kappa B alpha.

We then examined whether HSR1101 had any impact on nuclear translocation of NF-κB and phosphorylation of IκBα in LPS-activated BV2 cells using Western blotting analysis. The LPS treatment significantly augmented the translocation of the NF-κB p65 subunit into the nucleus, whereas the LPS-induced NF-κB translocation was dramatically inhibited by HSR1101 (Figure 5A for cytosolic NF-κB and Figure 5B for nuclear NF-κB). The inhibitory effect of HSR1101 on the nuclear translocation of NF-κB was further confirmed by immunocytochemical analysis. In vehicle-treated control cells, NF-κB p65 was mostly localized in the cytoplasm. In contrast, immunofluorescence staining of NF-κB p65 was increased in the nucleus of LPS-treated cells. HSR1101 treatment markedly suppressed the LPS-induced nuclear translocation of NF-κB, as indicated by arrows (Figure 5C). Furthermore, it was shown that LPS treatment enhanced the phosphorylation of IκBα, which was concentration-dependently suppressed by HSR1101 (Figure 5D). These results indicate that HSR1101 suppresses the nuclear translocation of NF-κB through inhibition of IκBα phosphorylation.

### 2.5. Effect of HSR1101 on LPS-Induced Cell Migration in BV2 Cells

**Figure 6:**
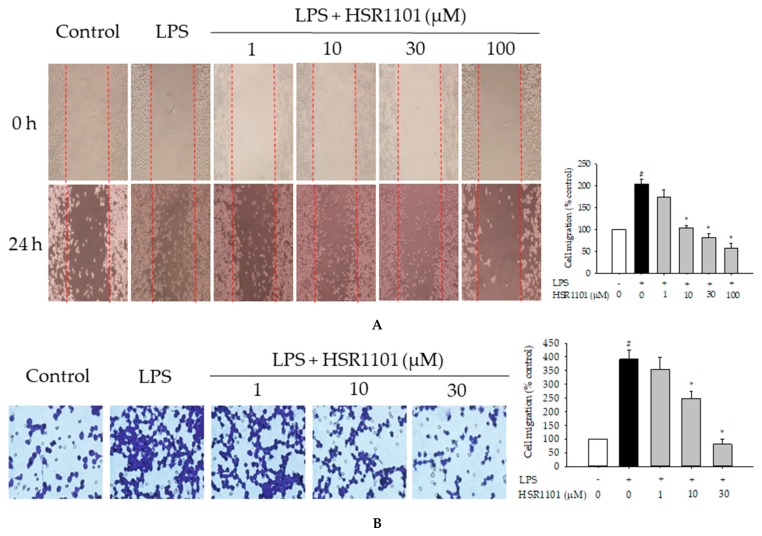
HSR1101 inhibited LPS-induced migration of BV2 cells. BV2 cells were co-treated with 1 μg/mL LPS and a series of concentrations of HSR1101 for 24 h and then analyzed for differences in migration of cells by wound healing (**A**) and transwell migration assays (**B**), as described in the Materials and Methods section. Data are expressed as mean ± SEM of at least three independent experiments. # *p* < 0.05 and * *p* < 0.05 vs the vehicle-treated control and LPS-treated groups, respectively. LPS, lipopolysaccharide.

It has been proved that the active migration of microglial cells is closely associated with the inflammatory responses [24,25]. Therefore, we then assessed whether HSR1101 could arrest LPS-stimulated migration of BV2 cells. Results revealed that LPS treatment markedly accentuated BV2 cell movement after 24 h of incubation in the wound healing and transwell migration assays. In these tests, LPS-stimulated cell migration was dramatically diminished by HSR1101 at the concentrations of 10 µM and above in both assays (Figure 6A,B).

### 2.6. Effect of HSR1101 on MAPK Phosphorylation in LPS-Treated BV2 Cells

**Figure 7:**
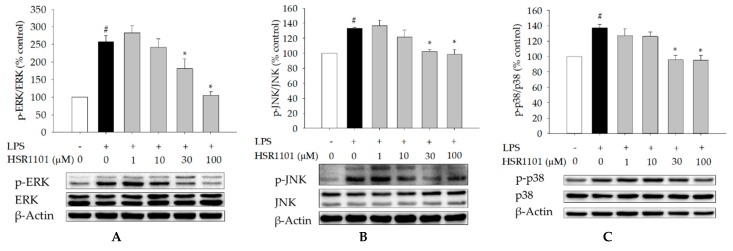
HSR1101 inhibited LPS-induced phosphorylation of the MAPK family including ERK1/2, JNK, and p38 MAPK in BV2 cells. BV2 cells were co-treated with 1 μg/mL LPS and a series of concentrations of HSR1101 for 24 h. Western blotting analyses were conducted using anti-phospho-ERK1/2 and anti-ERK1/2 antibodies (**A**), anti-phospho-JNK and anti-JNK antibodies (**B**), anti-phospho-p38 MAPK and anti-p38 MAPK antibodies (**C**), as described in the Materials and methods section. β-Actin was used for normalizing phospho-ERK1/2, phospho-JNK, and phospho-p38 MAPK. Representative blots are displayed. Data are expressed as mean ± SEM of at least three independent experiments. # *p* < 0.05 and * *p* < 0.05 vs the vehicle-treated control and LPS-treated groups, respectively. LPS, lipopolysaccharide; MAPK, mitogen-activated protein kinase; ERK1/2, extracellular signal-regulated kinase 1/2; JNK, c-Jun N-terminal kinase.

The MAPK family, which includes ERK1/2, JNK, and p38 MAPK, is thought to play pivotal roles in modulating pro-inflammatory mediators and cell migration in various cell types including microglial cells [20,21,22,23,26,27]. Therefore, we aimed to evaluate whether MAPK pathways were associated with anti-inflammatory and anti-migratory activities of HSR1101 in BV2 cells. It was revealed that treatment with LPS significantly increased the phosphorylation of ERK1/2, JNK and p38 MAPK and HSR1101 abated the LPS-induced phosphorylation of MAPKs (Figure 7).

### 2.7. Effects of MAPK Inhibitors on LPS-Induced Pro-Inflammatory Mediators and Cell Migration in BV2 Cells

The roles of MAPKs in the production of LPS-induced pro-inflammatory mediators have been well documented [20,28,29,30]. To elucidate the involvement of MAPKs in the inhibition of pro-inflammatory mediators by HSR1101, we investigated the effects of MAPK inhibitors on the production of pro-inflammatory mediators in LPS-treated BV2 cells. In consistence with the findings reported previously [20,28,29,30], we also observed in our study that U0126, SP600125 and SB203580, the specific inhibitors of MEK1/2 (a type of MAPK/ERK kinase), JNK and p38 MAPK, respectively, noticeably inhibited LPS-promoted production of IL-6, TNF-α, and NO (data not shown). Furthermore, we found that these MAPK inhibitors significantly attenuated iNOS and COX-2 expression and inhibited nuclear translocation of NF-κB and phosphorylation of IκBα in the LPS-treated BV2 cells (data not shown). Collectively, our observation confirmed that MAPKs/NF-κB pathway is involved in production of pro-inflammatory mediators in LPS-treated BV2 cells.

**Figure 8:**
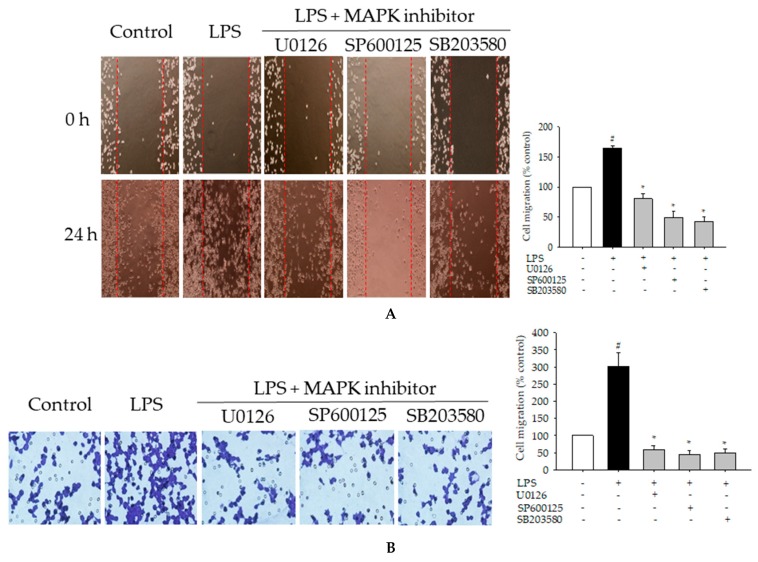
Specific MAPK inhibitors inhibited cell migration in BV2 cells. BV2 cells were co-treated with 1 μg/mL LPS and 10 μM of the specific MAPK inhibitors (U0126, SP600125, and SB203580) for 24 h, and cell migration was evaluated by wound healing (**A**) and transwell migration assays (**B**), as described in the Materials and methods section. Differences in BV2 cell migration were determined using ImageJ version 1.49 software. Data are expressed as mean ± SEM of at least three independent experiments. # *p* < 0.05 and * *p* < 0.05 vs the vehicle-treated control and LPS-treated groups, respectively. LPS, lipopolysaccharide; MAPK, mitogen-activated protein kinase.

To further elucidate the role of MAPKs in the anti-migratory effects of HSR1101, we examined the impacts of U0126, SP600125 and SB203580 on the LPS-induced cell migration. The results illustrated that these MAPK inhibitors dramatically diminished the LPS-promoted migration of BV2 cells in both wound healing and transwell migration assays (Figure 8A,B).

Based on our findings and previous reports, it is speculated that MAPKs may be the upstream kinases that trigger nuclear translocation of NF-κB through phosphorylation of IκBα in LPS-stimulated BV2 cells, and that HSR1101 exerts anti-inflammatory and anti-migratory effects through inhibition of MAPKs/ NF-κB pathway in BV2 cells.

## 3. Discussion

Cumulative evidence has suggested that microglial activation is associated with neuroinflammation and pathology of various neurodegenerative diseases [1,2,3,7,8,31]. Neuroinflammation can be stimulated and exacerbated by the over-production of pro-inflammatory mediators and cytokines by microglial cells, which eventually lead to neurodegeneration and cell death [32,33]. Therefore, the inhibition of pro-inflammatory mediators produced by activated microglia represents an attractive therapeutic approach to attenuate the progression of neuroinflammation and neurodegeneration. The current study demonstrated that HSR1101, an isoquinoline-1-carboxamide derivative with the *N*-hydroxyphenyl substituent, attenuated LPS-induced production of pro-inflammatory mediators (IL-6, TNF-α, and NO) and reversed LPS-suppressed anti-inflammatory cytokine (IL-10). Inhibition of iNOS and COX-2 may mediate, at least in part, the anti-inflammatory effect of HSR1101. We also found that HSR1101 effectively inhibited LPS-induced BV2 cell migration. Furthermore, the present study revealed that HSR1101 exerted the anti-inflammatory and anti-migratory effects through inhibition of the MAPKs/NF-κB signaling pathway.

In the present study, the newly synthesized isoquinoline-1-carboxamide derivatives with *N*-hydroxyphenyl, *N*-methoxyphenyl, *N*-(trifluoromethyl)phenyl, *N*-bis(trifluoromethyl)phenyl or *N*-chlorophenyl substituents (Figure 1) were evaluated for their effects on inflammation and cell migration using LPS-treated BV2 microglial cells as a model. Among the eleven compounds synthesized, five derivatives with *N*-hydroxyphenyl (HSR1101~1103) and *N*-methoxyphenyl (HSR1105 and 1106) substituents showed no cytotoxicity (Figure 2), and so were subsequently examined for their impact on the production of pro-inflammatory mediators. TNF-α is produced by stimulated astrocytes and microglia and serves as a strong etiologic factor in many neurological disorders [34,35]. In addition, IL-6 has been proved to have a direct pathogenic impact on neuroinflammatory, infectious, and neurodegenerative CNS diseases [36]. Although NO, a highly reactive gaseous signaling molecule, has both physiological and pathological functions in mediating interactions between glial cells and neurons in the CNS, over-production of NO mostly by iNOS during inflammatory processes is also known to contribute to a series of neurodegenerative pathologies [37,38]. The levels of these pro-inflammatory mediators were intensely enhanced by the LPS treatment of BV2 cells, being consistent with the previous reports [9,10,20,39,40]. Among the five selected compounds, the compounds with the *N*-hydroxyphenyl substituent (HSR1101~1103) exerted more potent inhibitory effects on the IL-6, TNF-α, and NO production (Figure 3A–C and Table 1). However, only HSR1101, not HSR1102 and 1103, showed the positive impact on the production of anti-inflammatory cytokine IL-10 (Figure 3D). IL-10 can modulate brain inflammatory settings and innate immune cells of the CNS [41]. In fact, the production of IL-10 is one of the most important mechanisms to counteract neuronal damage driven by excessive inflammation. Meanwhile, the decreased level of IL-10 has been reported to contribute to the neuroinflammatory process, which favors the development of neurodegenerative diseases including AD [42]. Our findings demonstrated that HSR1101 exerts anti-inflammatory action through both inhibiting pro-inflammatory mediators and inducing anti-inflammatory cytokine(s), suggesting a critical role of the hydroxyl moiety at the R1 position (Figure 1). It would be highly intriguing to investigate the molecular mechanisms by which HSR1101 inhibits or induces pro- or anti-inflammatory mediators, respectively.

It was also found that HSR1101 suppressed LPS-stimulated expression of iNOS and COX-2 in BV2 cells (Figure 4). iNOS is not expressed in healthy, uninjured tissue. Instead, its expression is induced in astrocytes and microglia under pathologic conditions including inflammation [43,44] Being an enzyme participating in NO synthesis, the expression of iNOS leads to delayed neuronal cell death, exacerbates glutamate neurotoxicity, and contributes to neurodevelopmental disorders [44]. Similarly, COX-2, an important enzyme required for the conversion of arachidonic acid to prostaglandins (PGs), is also highly induced by inflammatory stimuli, being the dominant source of PGs in a wide variety of diseases including inflammation and cancer [45]. In addition, the excessive activation of COX-2 can induce neuronal apoptosis, cognitive deficits and neurodegeneration [46,47]. Therefore, inhibition of iNOS and COX-2 expression by HSR1101 may provide benefits to attenuate neuroinflammation as well as neurodegeneration.

It is well-known that NF-κB is a nuclear transcription factor, playing a crucial role in the inflammatory responses mediated by microglia. In the resting state, the inactive form of NF-κB is present in the cytoplasm in complex with IκBs. The phosphorylation of IκBs, particularly IκBα, by IκB kinase is a critical step in the NF-κB activation. When cells are stimulated, IκB is phosphorylated and subsequently undergoes rapid degradation. This process triggers the release of NF-κB from the NF-κB–IκB complex, allowing it to translocate to the nucleus and then induce transcription of pro-inflammatory genes [19,48]. In the present study, LPS treatment led to the marked enhancement of IκBα phosphorylation and subsequent nuclear translocation of NF-κB p65 subunit in BV2 cells, being consistent with previous findings [9,17,20]. HSR1101 inhibited the LPS-induced IκBα phosphorylation as well as nuclear translocation of NF-κB (Figure 5). Accordingly, our findings indicate that HSR1101 inhibits LPS-induced inflammation by attenuating NF-κB translocation through suppression of IκBα phosphorylation.

Intriguingly, being attracted by factors secreted from damaged cells, microglial cells are activated and recruited toward the infected or damaged sites, where they are involved in both degenerative and regenerative responses as well as in the phagocytic clearance of cell debris [11]. Therefore, it is well-recognized that cell migration is closely associated with the activation of microglia cells, which leads to tissue damage and chronic inflammation [11,12]. Growing evidence has shown that, in addition to the pro-inflammatory mediators, increased production of NO can also positively modulate the migration of microglial cells during inflammatory processes in the CNS [49,50]. In light of the finding that HSR1101 remarkably inhibited inflammatory mediators including NO (Figure 3 and Table 1), we next examined whether it might impact on the LPS-induced migration of BV2 cells. As expected, HSR1101 dramatically reduced LPS-stimulated cell migration in both wound healing and transwell migration assays (Figure 6). Such findings strongly suggest that HSR1101 can abolish LPS-stimulated cell migration, and thereby alleviate neuroinflammatory processes.

MAPKs can induce various transcription factors that modulate immune responses such as neuroinflammation, consequently contributing to the pathogenesis of several neurodegenerative diseases such as PD and AD [51,52,53]. To further evaluate the intracellular signaling pathways involved in the anti-inflammatory and anti-migratory effects of HSR1101, we evaluated whether this compound had any influence on the MAPK pathways. In this study, LPS treatment of BV2 cells prominently enhanced phosphorylation of ERK1/2, JNK, and p38 MAPK. HSR1101 exerted significant inhibition of the phosphorylation of all three types of MAPKs (Figure 7). In accordance with these findings and previous reports [20,28,29,30], U0126, SP600125, and SB203580, the specific inhibitors of MEK1/2, JNK, and p38 MAPK, respectively, suppressed the LPS-induced production of IL-6, TNF-α, and NO (data not shown). Moreover, LPS-induced cell migration was also reduced by these specific MAPK inhibitors (Figure 8). Furthermore, in agreement with the previous findings [54], we also confirmed that all three MAPK inhibitors effectively reduced the LPS-induced IκBα phosphorylation and nuclear translocation of NF-κB, suggesting that the MAPK pathways may serve as upstream kinases to induce the phosphorylation of IκBα, which subsequently leads to the nuclear translocation of NF-κB during the inflammatory process.

Recent studies have demonstrated key functions of the Toll-like receptor (TLR) family in the innate and adaptive immune system, especially in the CNS [55]. The activation of TLRs on microglia may contribute to initiating inflammation in the CNS and the development of neurodegenerative diseases [56]. It is known that LPS can stimulate and activate TLR-4, a member of the TLR family, expressed on the surface of microglia. Upon the activation of TLR-4 by LPS, an intracellular adapter protein, MyD88, is recruited and, subsequently, both the NF-κB and MAPKs pathways are activated, producing various pro-inflammatory mediators [57]. Thus, inhibition of TLR-4-mediated signaling molecule(s) may be beneficial to counteract inflammation. It is demonstrated in this study that HSR1101 inhibited both phosphorylation of MAPKs and nuclear translocation of NF-κB. It would be worthwhile to further elucidate the upstream signaling molecules of MAPKs to establish exact mechanism(s) mediating the anti-inflammatory and anti-migratory actions of HSR1101.

**Figure 9:**
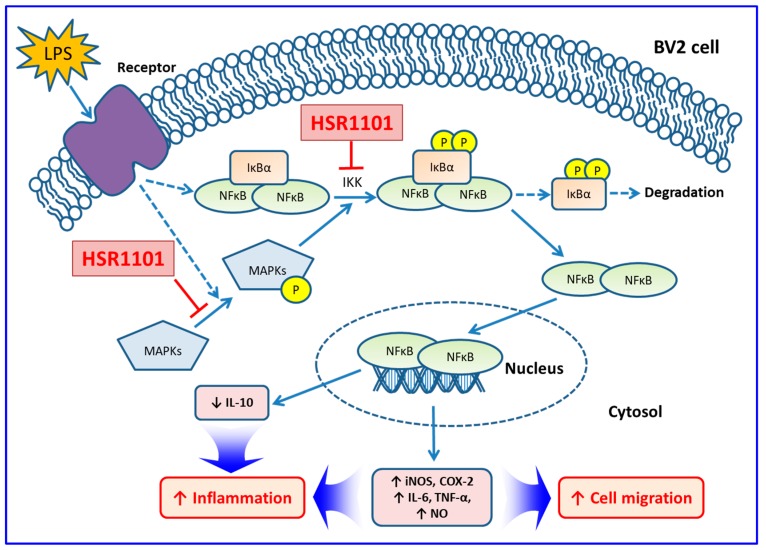
A schematic diagram showing the signaling pathways involved in anti-inflammatory and anti-migratory actions by HSR1101 in BV2 microglial cells. Solid arrows indicate the pathway(s) examined, while dotted arrows indicate the pathway(s) not examined in this study. LPS, lipopolysaccharide; NF-κB, nuclear factor-kappa B; IκBα, inhibitor of kappa B alpha; IKK, inhibitor of kappa B kinase; MAPKs, mitogen-activated protein kinases; iNOS, inducible nitric oxide synthase; COX-2, cyclooxygenase-2; IL-6, interleukin-6; TNF-α, tumor necrosis factor-alpha; NO, nitric oxide; IL-10, interleukin-10, P, phosphorylation.

Based on our findings in this study, a schematic diagram indicating the signaling pathways involved in the anti-inflammatory and anti-migratory actions of HSR1101 is shown in Figure 9. Among newly synthesized isoquinoline-1-carboxamide derivatives tested in this study, HSR1101 was a potent and promising compound, suppressing LPS-induced inflammation and cell migration in BV2 microglial cells without showing notable cytotoxicity. Our results also indicated that inhibition of the MAPKs/NF-κB pathway was involved in the anti-inflammatory and anti-migratory effects of HSR1101. Collectively, HSR1101 may have beneficial impacts on various neurodegenerative disorders associated with neuroinflammation and microglial activation.

## 4. Materials and Methods

### 4.1. Chemicals and Reagents

Fetal bovine serum (FBS) and antibiotic–antimycotic agents were provided by Gibco BRL (Grand Island, NY, USA). Dimethyl sulfoxide (DMSO), LPS (*Escherichia coli*, serotype 011:B4), 3-(4,5-dimethyldiazol-2-yl)-2,5-diphenyltetrazolium bromide (MTT), and anti-β-actin antibody were bought from Sigma–Aldrich (St. Louis, MO, USA). U0126 and antibodies specifically recognizing iNOS, COX-2, NF-κB p65, IκBα, and non-phosphorylated or phosphorylated ERK1/2, JNK, and p38 MAPK were purchased from Cell Signaling Technology (Danvers, MA, USA). Anti-phospho-IκBα (S36) antibody and SB203580 were supplied by Abcam (Cambridge, MA, USA). SP600125 was provided by Calbiochem (Darmstadt, Germany). Dulbecco’s modified Eagle’s medium (DMEM), Alexa Fluor 488-conjugated anti-mouse IgG, and 4′,6 diamidino-2-phenylindole dihydrochlorides (DAPI) were supplied by Thermo Scientific (Rockford, IL, USA).

### 4.2. Synthesis of Isoquinoline-1-carboxamide Derivatives

**Scheme 1:**
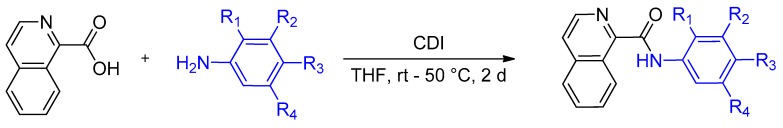
Synthesis of isoquinoline-1-carboxamide derivatives.

*N*-substitutedphenylisoquinoline-1-carboxamides (HSR1101~1111) were prepared by the simple amidation reaction. The starting isoquinoline-1-carboxylic acid was treated with various substituted anilines in the presence of the coupling reagent 1,1′-carbonyldiimidazole (CDI in Scheme 1) in tetrahydrofuran at room temperature to give a series of isoquinoline-1-carboxamide derivatives. The purity of the synthesized compounds was more than 99% with a yield of 43%–89% as indicated in Figure 1. Spectral data of isoquinoline-1-carboxamide derivatives (HSR1101~1111) are provided in the Supplementary Materials.

All compounds were dissolved in DMSO to prepare 10 mM stock solutions and then serially diluted before use to obtain a series of concentrations tested in this study.

### 4.3. Cell Culture, Treatments, and Measurements of Cell Viability

BV2 microglial cells were cultured in DMEM solution containing 1% antibiotics and 10% FBS at 37 °C in an incubator with 5% CO_2_. The cells were then seeded into 24-well plates at a density of 2.5 × 10^5^ cells/well and incubated at 37 °C for 24 h. To determine whether the synthesized isoquinoline-1-carboxamides exhibited any cytotoxicity in BV2 cells, the cells were treated with the tested compounds at 30 and 100 μM. Simultaneously, control cells were treated with serum-free media containing 1% DMSO instead. After treatment for 24 h, MTT assay was performed, as previously described [9] with minor modification. Briefly, the cells were incubated with MTT solution at a final concentration of 0.5 mg/mL at 37 °C for 3 h, and the supernatants were carefully removed. DMSO was then added to each well to dissolve the formazan crystals, and the absorbance was measured at 550 nm with a microplate reader (SpectraMax M2^e^, Molecular Devices, Sunnyvale, CA, USA). The cell viability was expressed as the percentage of absorbance determined for vehicle-treated control cells.

### 4.4. Measurements of IL-6, TNF-α, NO, and IL-10

BV2 cells were seeded into 24-well plates at a density of 2.5 × 10^5^ cells/well and incubated at 37 °C for 24 h. To evaluate the effects of the synthesized isoquinoline-1-carboxamides on LPS-induced pro-inflammatory mediators, the cells were co-treated with 1 µg/mL LPS and the tested compounds at the indicated concentrations in serum-free media for 24 h. Control cells were treated with serum-free media containing 1% DMSO instead. After the treatment, the culture media were gently collected and centrifuged at 1,500 rpm at 4 °C for 10 min, and the supernatants were collected and stored at −80 °C until used. The levels of IL-6, TNF-α, and IL-10 were measured by enzyme-linked immune sorbent assay (ELISA) kits (Koma Biotech, Seoul, Korea), according to the manufacturer’s instructions. The concentrations of NO were determined using Griess reagent (Promega Corporation, Madison, WI, USA), as described previously [10,20]. The levels of IL-6, TNF-α, NO, and IL-10 were then calculated from their respective standard reference curves generated simultaneously.

### 4.5. Western Blotting

BV2 cells were seeded into 35 mm culture dishes at a density of 1 × 10^6^ cells/dish and incubated at 37 °C for 24 h. The cells were co-treated with 1 µg/mL LPS and the determined concentration of the tested compounds for 24 h. Control cells were treated with serum-free media containing 1% DMSO instead. After the treatment, the cells were gently washed with cold phosphate-buffered saline (PBS) and lysed with lysis buffer [10 mL of lysis buffer contained 150 mM sodium chloride, 10 mM Tris-HCl (pH 7.5), 4.5 mM sodium pyrophosphate, 2 mM ethylenediaminetetraacetic acid, 0.5% octylphenoxy poly(ethyleneoxy)ethanol (IGE-PAL CA-630), 1% (v/v) Triton X-100, 1 mM sodium fluoride, 1 mM sodium orthovanadate, 10 mM β-glycerophosphate, and one tablet of protease inhibitor cocktail (Roche Diagnostic GmbH, Mannheim, Germany)] on ice for 30 min. Cell lysates were centrifuged at 16,000 rpm at 4 °C for 30 min. The supernatants containing the extracted proteins were collected and stored at −80 °C until used. To elucidate the effect of the tested compounds on the nuclear translocation of NF-κB, cytosolic and nuclear extractions were separately prepared by using NE-PER^®^ Nuclear and Cytoplasmic Extraction Reagents (Thermo Fisher, Rockford, IL, USA). Electrophoresis and immunoblotting were performed according to previously reported procedures [9,20]. Specifically, cell lysates, each containing the equivalent amounts of proteins were resolved by SDS-PAGE and transferred to nitrocellulose membranes at 100 V for 90 min. The transferred membranes were then blocked with 5% skim milk (BD Biosciences, San Jose, CA, USA) at room temperature for 90 min and incubated with primary antibodies in bovine serum albumin (MP Biomedicals, Solon, OH, USA) at 4 °C overnight. The membranes were rinsed with Tris-buffered saline containing 0.1% Tween 20 and incubated with horseradish peroxidase-conjugated secondary antibodies at room temperature for 90 min. Specific bands were detected by the Bio-Rad ChemiDoc XRS imaging system with enhanced chemiluminescent reagents (Bio-Rad, Hercules, CA, USA).

### 4.6. Immunocytochemistry

To elucidate the intracellular localization of NF-κB, immunocytochemistry was conducted according to the procedures reported previously [20,58]. Briefly, BV2 cells were cultured for 24 h at a density of 5 × 10^4^ cells/well on the coverslips placed in the wells of 24-well plates and co-treated with 1 µg/mL LPS and 30 μM HSR1101 in serum-free media. Control cells were treated with serum-free media containing 0.3% DMSO instead. After treating for 24 h, the cells were fixed with 3.7% paraformaldehyde for 15 min and gently washed three times with PBS. The fixed cells were then permeabilized in 1% Triton X-100 for 5 min and gently washed three times with PBS. After blocking non-specific bindings with 5% goat serum for 30 min, the cells were incubated with anti-NF-κB p65 antibody at a dilution of 1:100 overnight at 4° C, followed by gentle washing with PBS three times. Afterward, the cells were incubated with fluorescent-conjugated secondary antibody at a dilution of 1:400 and counterstained with DAPI at a dilution of 1:400 at room temperature for 1 h in the dark. After gentle rinsing with PBS, the coverslips were carefully mounted with Dako Fluorescent Mounting Medium (Dako, Carpinteria, CA, USA). Fluorescent signals were visualized under a Nikon confocal laser-scanning microscope (Nikon Instruments Inc., Melville, NY, USA).

### 4.7. Cell Migration Assays

#### 4.7.1. Wound Healing Assay

BV2 cells were seeded into 24-well plates at a density of 2.5 × 10^5^ cells/well and incubated at 37 °C for 24 h. To assess the migration of cells, the cells were equally wounded with a sterile 100-μL pipette tip [26,59,60]. To evaluate how the tested compounds affected the LPS-induced cell mobility, the cells were treated with 1 µg/mL LPS with and without a series of concentrations of HSR1101 or 10 µM of MAPK inhibitors for 24 h. Simultaneously, control cells were treated with serum-free media containing 1% DMSO instead. The relative cell migration during 24 h were determined by measuring the changes in the area of the wounds at 0 and 24 h under a Nikon phase-contrast microscope (Nikon Instruments Inc., Melville, NY, USA) [60], using ImageJ version 1.49 software obtained from National Institutes of Health homepage (https://imagej.nih.gov/ij/). The cell migration was expressed as the percentages of the respective vehicle-treated control cells.

#### 4.7.2. Transwell Migration Assay

BV2 cells were plated at a density of 5 × 10^4^ cells/well into the inserts (6.5 mm in diameter with an 8.0-µm pore size membrane) of a Costar transwell system (Corning Incorporated, Kennebunk, ME, USA). After incubation at 37 °C for 24 h, the bottom wells were filled with 1 µg/mL LPS in serum-free media with or without a series of concentrations of HSR1101 or 10 µM of MAPK inhibitors. After treating for 24 h, non-migrated cells were gently removed and the migrated cells were fixed with 3.7% paraformaldehyde for 20 min, then permeabilized with methanol for 10 min, followed by staining with 0.5% crystal violet for 10 min, as described elsewhere [20,26,59]. The numbers of cells that migrated through the membrane were counted from four random fields of each well under a Nikon phase-contrast microscope (Nikon Instruments Inc., Melville, NY, USA). Cell migration was expressed as the percentages of the respective vehicle-treated control cells.

### 4.8. Statistical Analysis

All data are presented as mean ± SEM of at least three independent measurements. Statistical significances were analyzed by one-way analysis of variance (ANOVA) using SigmaPlot 12.5 software (Systat Software Inc., San Jose, CA, USA). Statistical significance was considered at *p* < 0.05. IC_50_ values were determined by non-linear regression using GraphPad Prism 5 (GraphPad Software Inc., La Jolla, CA, USA).

## 5. Conclusions

Eleven compounds of isoquinoline-1-carboxamide derivatives (HSR1101~1111) with *N*-hydroxyphenyl, *N*-methoxyphenyl, *N*-(trifluoromethyl)phenyl, *N*-bis(trifluoromethyl)phenyl or *N*-chlorophenyl substituents were assessed for their anti-inflammatory and anti-migratory effects using LPS-activated BV2 microglial cells. Among the tested compounds, *N*-(2-hydroxyphenyl)isoquinoline-1-carboxamide (HSR1101) exhibited a potent anti-inflammatory effect through inhibition of LPS-induced production of pro-inflammatory mediators such as IL-6, TNF-α, and NO and the reversal of the LPS-suppressed anti-inflammatory cytokine IL-10. Moreover, HSR1101 repressed expression of iNOS and COX-2 as well as cell migration in LPS-stimulated BV2 cells. We found that inhibition of the MAPKs/NF-κB signaling pathway was involved in the anti-inflammatory and anti-migratory activities of HSR1101 in LPS-treated BV2 cells. Based on our findings, HSR1101 may be considered as a promising therapeutic candidate to prevent or treat various neurodegenerative diseases closely associated with neuroinflammation and microglial activation.

## Figures and Tables

**Table 1:** IC_50_ values of isoquinoline-1-carboxamide derivatives inhibiting IL-6, TNF-α, or NO production in LPS-treated BV2 microglial cells.

Pro-Inflammatory Mediators	IC_50_ Value ^a^				
	HSR1101	HSR1102	HSR1103	HSR1105	HSR1106
**IL-6**	39.13	29.52	30.55	56.59	76.38
**TNF-α**	29.18	25.92	70.17	71.66	67.81
**NO**	22.84	28.52	22.42	42.27	75.13

**^a^**: IC_50_ value represents the concentration of each compound inhibiting by 50% of LPS-induced IL-6, TNF-α, or NO. LPS, lipopolysaccharide; IL, interleukin; TNF-α, tumor necrosis factor-alpha; NO, nitric oxide.

## Supplementary Materials

Supplementary materials can be found at https://www.mdpi.com/1422-0067/21/7/2319/s1.

## Abbreviations

AD	Alzheimer’s disease
CNS	Central nervous system
COX-2	Cyclooxygenase-2
ERK1/2	Extracellular signal-regulated kinase 1/2
IκBs	Inhibitors of kappa B
IL	Interleukin
iNOS	Inducible nitric oxide synthase
JNK	C-Jun N-terminal kinase
LPS	Lipopolysaccharide
MAPK	Mitogen-activated protein kinase
NF-κB	Nuclear factor-kappa B
NO	Nitric oxide
PD	Parkinson’s disease
TLR	Toll-like receptor
TNF-α	Tumor necrosis factor-alpha
