# Supplementary material for: Anti-Inflammatory and Anti-Migratory Activities of Isoquinoline-1-Carboxamide Derivatives in LPS-Treated BV2 Microglial Cells via Inhibition of MAPKs/NF-κB Pathway
Source: Int J Mol Sci. 2020 Mar 27;21(7):2319. doi: 10.3390/ijms21072319 (PMC7177615; doi:10.3390/ijms21072319)

Supporting Information

General Information

All the reactions were carried out in oven dried glassware with freshly distilled dry solvents under anhydrous conditions unless otherwise indicated and all commercial chemicals were used as obtained. Evaporation of organic solutions was achieved by rotary evaporation with a water bath temperature below 40 °C. All the products obtained were purified by column chromatography using silica gel (100-200 mesh). Thin layer chromatography was performed on E Merck silica gel GF-254 pre-coated plates; identification was performed under UV illumination. ^1H and ^{13}C NMR were recorded in JEOL 400 and 101 MHz spectrometer respectively. The chemical shifts are reported in ppm downfield to TMS ($\delta = 0$) for ^1H NMR and relative to the central $CDCl_3$ resonance ($\delta = 77.0$) for ^{13}C NMR. Data are reported as follows: chemical shift in ppm (d), multiplicity (s = singlet, d = doublet, t = triplet, m = multiplet), coupling constant (Hz), and integration. High resolution mass spectra (HRMS) recorded on LCQ Fleet-Thermo Scientifics.

General Procedure for the synthesis of *N*-substitutedphenylisoquinoline-1-carboxamide derivatives (HSR1101 - HSR1111).

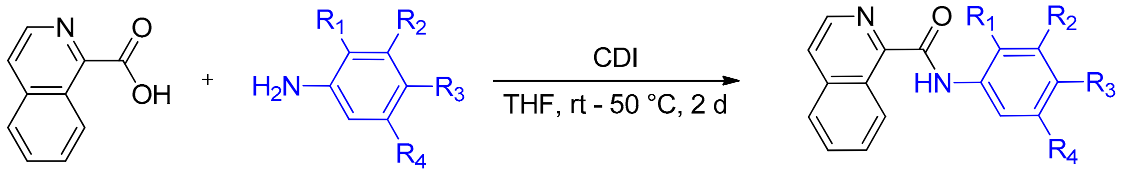

To a 0.5 M solution of isoquinoline-1-carboxylic acid (1.0 eq.) in THF, substitutedaniline (2.0 eq.) and 1,1'-carbonyldiimidazole (CDI) (1.2 eq.) were added. The mixture was stirred at room temperature for 6 h. The mixture was poured into water (50 mL)

and extracted with EtOAc (3 x 20 mL). The organic layer was dried over $MgSO_4$. The organic layer was concentrated *in vacuo* to afford the corresponding *N*-substitutedphenylisoquinoline-1-carboxamides (**HSR1101** - **HSR1111**).

***N*-(2-Hydroxyphenyl)isoquinoline-1-carboxamide** (**HSR1101**)

Brown solid; 47% yield ; ^{1}H NMR (400 MHz, DMSO-*d6*) δ (ppm) 10.67 (s, 1H), 10.29 (s, 1H), 9.50 (d, 1H, J = 7.8 Hz), 8.66 (d, 1H, J = 5.5 Hz), 8.41 (m, 1H), 8.15 (d, 1H, J = 5.5 Hz), 8.10 (d, 1H, J = 8.2 Hz), 7.89 – 7.79 (m, 2H), 7.02 – 6.97 (m, 2H), 6.91 – 6.87 (m, 1H); ^{13}C NMR (100 MHz, DMSO- *d6*) δ (ppm) 163.5, 148.3, 147.4, 141.0, 137.7, 131.4, 129.5, 127.9, 127.3, 126.8, 126.8, 125.4, 124.8, 112.0, 119.8, 115.4. HRMS m/z $[M+H]^+$ calculated for $C_{16}H_{13}N_2O_2$: 265.0972; Found: 265.0963.

***N*-(3-Hydroxyphenyl)isoquinoline-1-carboxamide** (**HSR1102**)

Yellow solid; 50% yield ; ^{1}H NMR (400 MHz, DMSO-*d6*) δ (ppm) 10.65 (s, 1H), 9.48 (s, 1H), 8.78 (d, 1H, J = 8.7 Hz), 8.62 (d, 1H, J = 6.0 Hz), 8.10 – 8.06 (m, 2H), 7.88 – 7.84 (m, 1H), 7.78 (m, 1H), 7.48 (t, 1H, J = 2.3 Hz), 7.24 (m, 1H), 7.17 (t, 1H, J = 8.2 Hz), 6.57 – 6.54 (m, 1H); ^{13}C NMR (100 MHz, DMSO- *d6*) δ (ppm) 165.2, 158.2, 152.4, 141.5, 140.3, 131.4, 130.0, 129.1, 127.8, 126.8, 123.9, 116.61, 113.3, 107.6. HRMS m/z $[M+H]^+$ calculated for $C_{16}H_{13}N_2O_2$: 265.0972; Found: 265.0957 .

***N*-(4-Hydroxyphenyl)isoquinoline-1-carboxamide** (**HSR1103**)

White solid; 43% yield ; ^{1}H NMR (400 MHz, DMSO-d6) δ (ppm) 10.57 (s, 1H), 9.34 (s, 1H), 8.83 (d, 1H, J = 8.2 Hz), 8.60 (d, 1H, J = 5.5 Hz), 8.08 (d, 1H, J = 8.2 Hz), 8.05 (d, 1H, J = 5.5 Hz), 7.84 (t, 1H, J = 7.3 Hz), 7.74 (t, 1H, J = 7.3 Hz), 7.68 – 7.65 (m, 2H), 6.79 – 6.77 (m,

2H); ^{13}C NMR (100 MHz, DMSO- d_6) δ (ppm) 164.7, 154.4, 152.4, 141.4, 137.1, 131.3, 130.9, 129.0, 127.8, 126.9, 126.0, 123.8, 122.2, 115.7. HRMS m/z $[M+H]^+$ calculated for $C_{16}H_{13}N_2O_2$: 265.0972; Found: 265.0956 .

***N*-(2-Methoxyphenyl)isoquinoline-1-carboxamide (HSR1104)**

Yellow solid; 77% yield ; ^1H NMR (400 MHz, $CDCl_3$) δ (ppm) 10.74 (s, 1H), 9.69 (d, 1H, J = 7.8 Hz), 8.68 (d, 1H, J = 7.8 Hz), 8.59 (dd, 1H, J = 5.5 Hz), 7.9 – 7.86 (m ,2H), 7.79 – 7.70 (m ,2H), 7.15 – 7.04 (m, 2H), 6.96 (d, 1H, J = 7.8 Hz), 3.98 (s, 3H); ^{13}C NMR (100 MHz, $CDCl_3$) δ (ppm) 149.0, 140.0, 137.8, 130.9, 129.0, 128.0, 127.8, 127.3, 127.1, 124.8, 124.1, 121.1, 119.8, 110.2, 56.0. HRMS m/z $[M+H]^+$ calculated for $C_{17}H_{15}N_2O_2$: 279.1128; Found: 279.1109.

***N*-(3-Methoxyphenyl)isoquinoline-1-carboxamide (HSR1105)**

Yellow solid; 73% yield ; ^1H NMR (400 MHz, $CDCl_3$) δ (ppm) 10.22 (s, 1H), 9.74 (d, 1H, J =7.8 Hz), 8.50 (d, 1H, J = 5.5 Hz), 7.88 – 7.82 (m, 2H), 7.68-7.56 (m, 4H), 6.97 – 6.93 (m, 2H), 3.83 (s, 3H),; ^{13}C NMR (100 MHz, $CDCl_3$) δ (ppm) 163.5, 160.3, 139.6, 139.3, 137.8, 131.0, 129.8, 129.2, 128.0, 127.1, 125.1, 112.2, 110.5, 105.3, 55.5. HRMS m/z $[M+H]^+$ calculated for $C_{17}H_{15}N_2O_2$: 279.1128; Found: 279.1103.

***N*-(4-Methoxyphenyl)isoquinoline-1-carboxamide (HSR1106)**

Yellow solid; 75% yield ; ^1H NMR (400 MHz, $CDCl_3$) δ (ppm) 10.4 (s, 1H), 9.72 (d, 1H, J = 8.7 Hz), 8.53 (d, 1H, J = 5.5 Hz), 7.92 – 7.88 (m, 2H), 7.80 – 7.72 (m, 2H), 7.61 (m, 1H), 7.31 – 7.30 (m, 2H), 6.73 – 6.71 (m, 2H); ^{13}C NMR (100 MHz, $CDCl_3$) δ (ppm) 163.6, 156.5, 147.8, 140.0, 137.7, 131.4, 130.7, 128.9, 128.0, 127.4, 127.0, 124.8, 121.6, 114.3, 55.6. HRMS m/z $[M+H]^+$ calculated for $C_{17}H_{15}N_2O_2$: 279.1128; Found: 279.1112.

***N*-(2-(Trifluoromethyl)phenyl)isoquinoline-1-carboxamide** (**HSR1107**)

White solid; 87% yield ; ^{1}H NMR (400 MHz, $CDCl_3$) δ (ppm) 10.56 (s, 1H), 9.24 (dd, 1H, J = 7.3 Hz), 8.14 – 8.08 (m, 2H), 7.43 – 7.39 (m, 2H), 7.29 – 7.24 (m, 2H), 7.23 – 7.14 (m, 2H), 6.78 (t, 1H, J = 8.7 Hz); ^{13}C NMR (100 MHz, $CDCl_3$) δ (ppm) 164.0, 146.8, 140.3, 137.8, 135.8, 132.9, 130.7, 129.2, 127.6, 127.5, 127.1, 125.3, 124.1, 123.2. HRMS m/z [M-H]$^{-}$ calculated for $C_{17}H_{10}F_3N_2O$: 315.0750; Found: 315.0767.

***N*-(3-(Trifluoromethyl)phenyl)isoquinoline-1-carboxamide** (**HSR11018**)

White solid; 85% yield ; ^{1}H NMR (400 MHz, $CDCl_3$) δ (ppm) 10.58 (s, 1H), 9.71 (d, 1H, J = 8.2 Hz), 8.53 (d, 1H, J = 6.0 Hz), 8.17 (s, 1H), 7.99 (d, 1H, J = 8.2 Hz), 7.90 – 7.88 (m, 2H), 7.79 – 7.72 (m, 2H), 7.52 (t, 1H, J = 7.8 Hz), 7.40 (d, 1H, J = 7.8 Hz); ^{13}C NMR (100 MHz, $CDCl_3$) δ (ppm) 163.8, 146.8, 139.8, 136.6, 137.8, 131.0, 129.7, 129.3, 127.7, 127.1, 125.4, 122.8, 120.8, 116.6. HRMS m/z [M-H]$^{-}$ calculated for : $C_{17}H_{10}F_3N_2O$: 315.0750; Found: 315.0770

***N*-(4-(Trifluoromethyl)phenyl)isoquinoline-1-carboxamide** (**HSR1109**)

White solid; 78% yield ; ^{1}H NMR (400 MHz, $CDCl_3$) δ (ppm) 10.61 (s, 1H), 9.71 (d, 1H, J = 7.3 Hz), 8.55 (d, 1H, J = 5.5 Hz), 7.96 (d, 2H, J = 8.2 Hz), 7.92 – 7.89 (m, 2H), 7.80 – 7.73 (m, 2H), 7.67 (d, 2H, J = 8.7 Hz) ; ^{13}C NMR (100 MHz, $CDCl_3$) δ (ppm) 163.9, 146.8, 141.1, 140.0, 137.8, 130.9, 129.3, 127.6, 127.1, 126.4, 126.4, 126.0, 125.4, 119.4. HRMS m/z [M+H]$^{+}$ calculated for : $C_{17}H_{12}F_3N_2O$: 317.0896; Found: 317.0869.

***N*-(3,5-Bis(trifluoromethyl)phenyl)isoquinoline-1-carboxamide** (**HSR1110**)

White solid; 89% yield ; ^{1}H NMR (400 MHz, $CDCl_3$) δ (ppm) 10.78 (s, 1H), 9.71 (m, 1H), 8.54 (d, 1H, J = 5.5 Hz), 8.34 (s, 2H), 7.92 – 7.90 (m, 2H), 7.80 – 7.73 (m, 2H), 7.65 (s, 1H); ^{13}C NMR (100 MHz, $CDCl_3$) δ (ppm) 163.9, 146.1, 139.8, 139.5, 137.8, 133.0, 132.7, 132.4, 131.1, 129.5, 127.5, 127.2, 125.8, 124.7, 121.9, 119.4, 117.4. HRMS m/z $[M-H]^-$ calculated for : $C_{18}H_9F_6N_2O$: 383.0624; Found: 383.0635.

***N*-(3-Chlorophenyl)isoquinoline-1-carboxamide (HSR1111)**

White solid; 63% yield ; ^{1}H NMR (400 MHz, $CDCl_3$) δ (ppm) 10.44 (s, 1H), 9.73 (d, 1H, J = 7.3 Hz), 8.53 (d, 1H, J = 5.5 Hz), 7.99 (t, 1H, J = 2.1 Hz), 7.91 – 7.88 (m, 2H), 7.80 – 7.72 (m, 2H), 7.65 (dd, 1H, J = 8.2 Hz), 7.34 (t, 1H, J = 7.8 Hz), 7.15 (dd, 1H, J = 8.2 Hz) ; ^{13}C NMR (100 MHz, $CDCl_3$) δ (ppm) 163.8, 147.0, 140.0, 139.3, 137.8, 134.9, 130.1, 129.2, 127.78, 127.4, 127.1, 125.3, 124.3, 119.9, 117.8. HRMS m/z $[M+H]^+$ calculated for : $C_{16}H_{12}ClN_2O$: 283.0633; Found: 283.0609.

Appendix

^{1}H, ^{13}C & Mass Spectral Copies of Tested Compounds in this Study

N-(2-Hydroxyphenyl)isoquinoline-1-carboxamide (HSR1101)

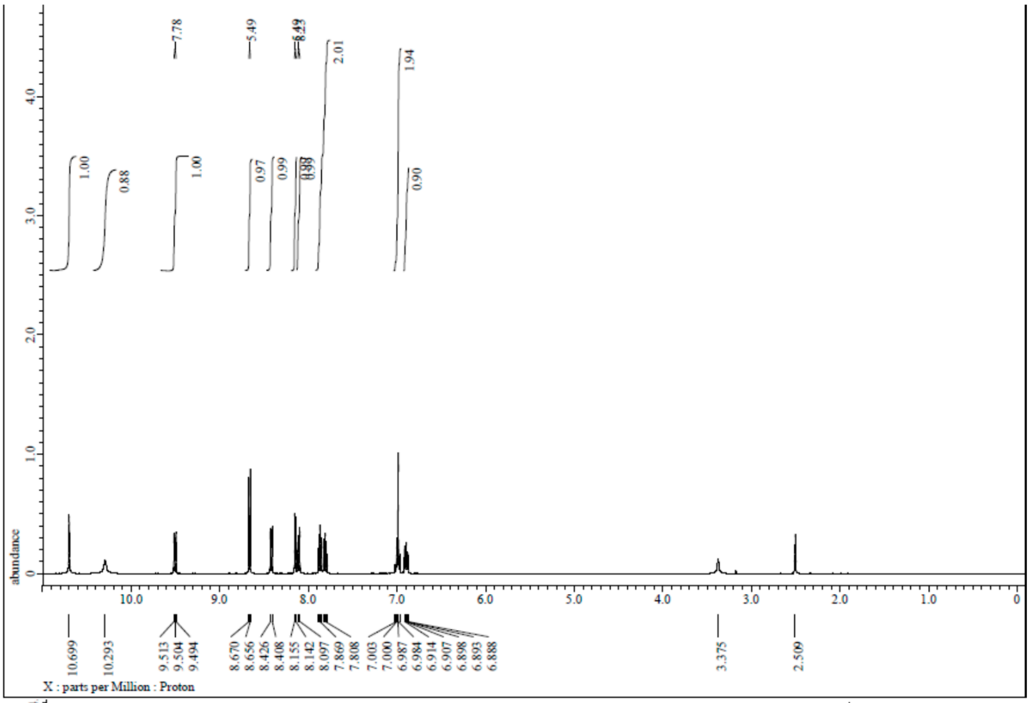

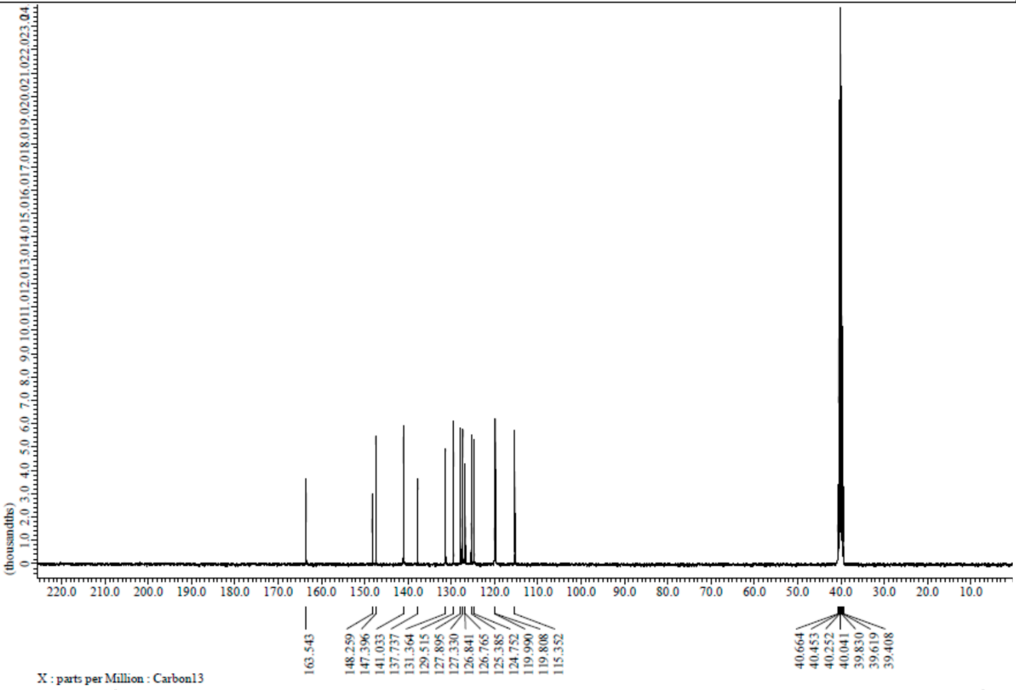

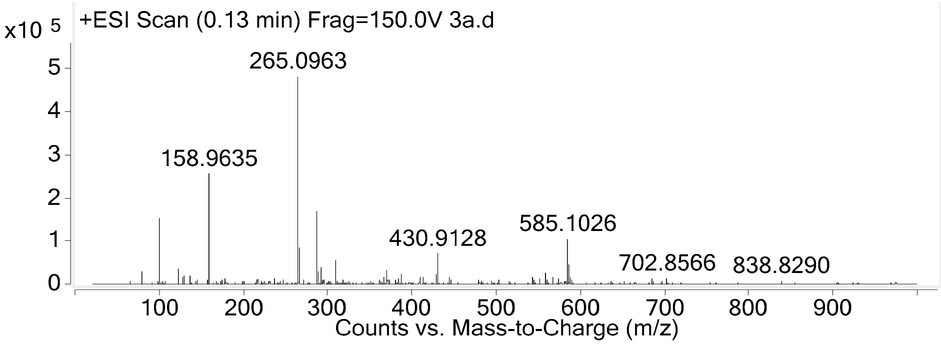

N-(3-Hydroxyphenyl)isoquinoline-1-carboxamide (HSR1102)

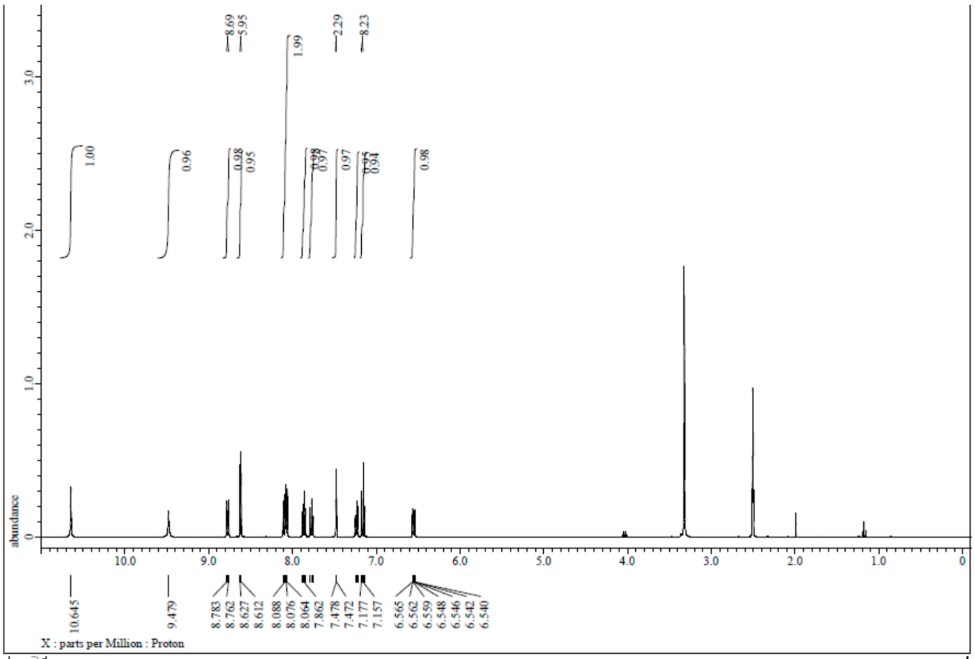

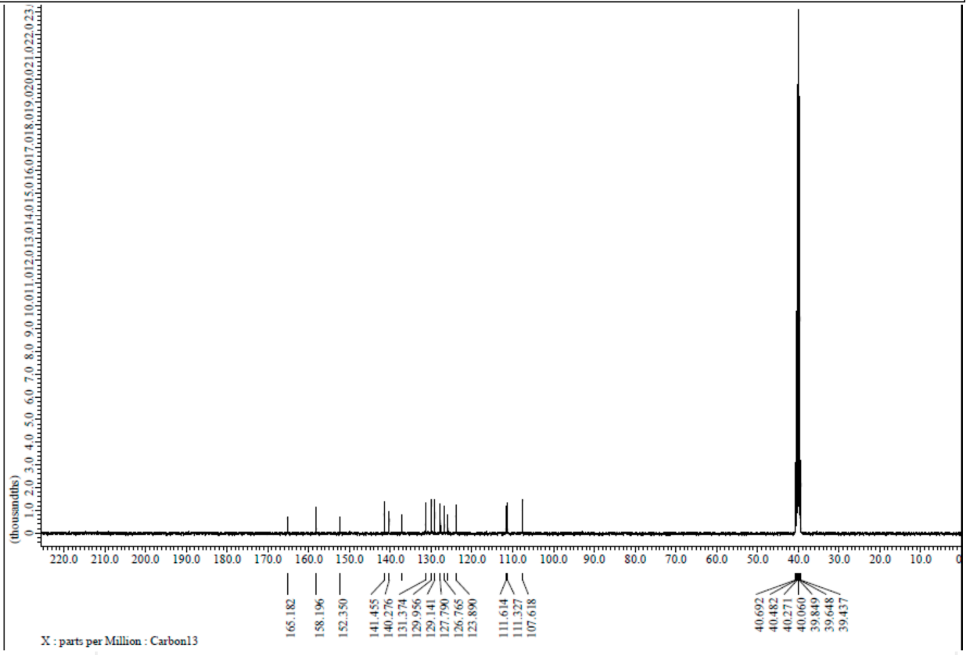

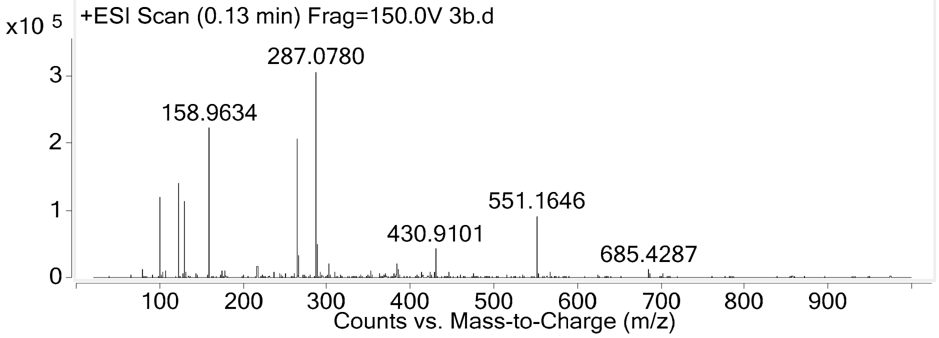

***N*-(4-Hydroxyphenyl)isoquinoline-1-carboxamide (HSR1103)**

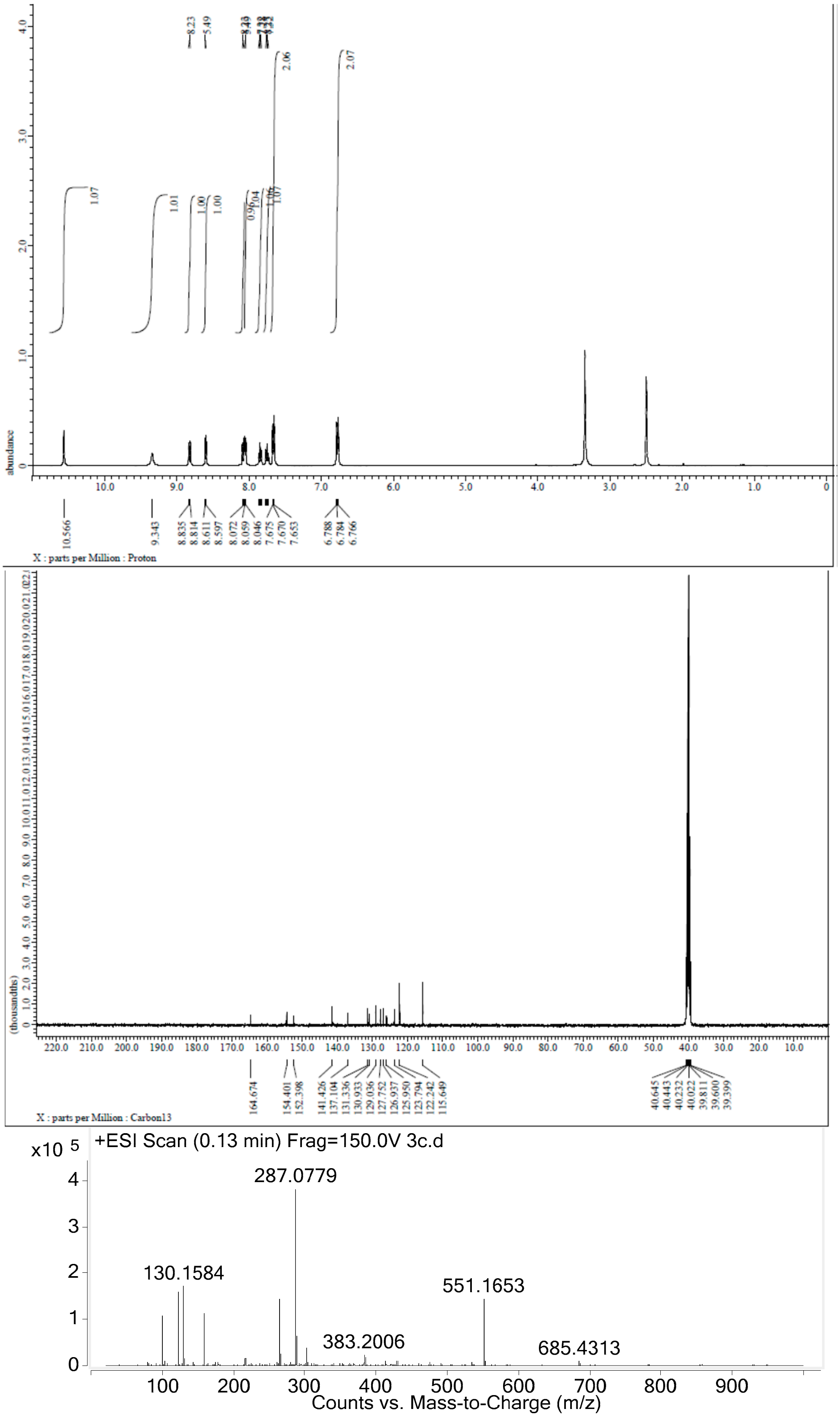

***N*-(2-Methoxyphenyl)isoquinoline-1-carboxamide (HSR1104)**

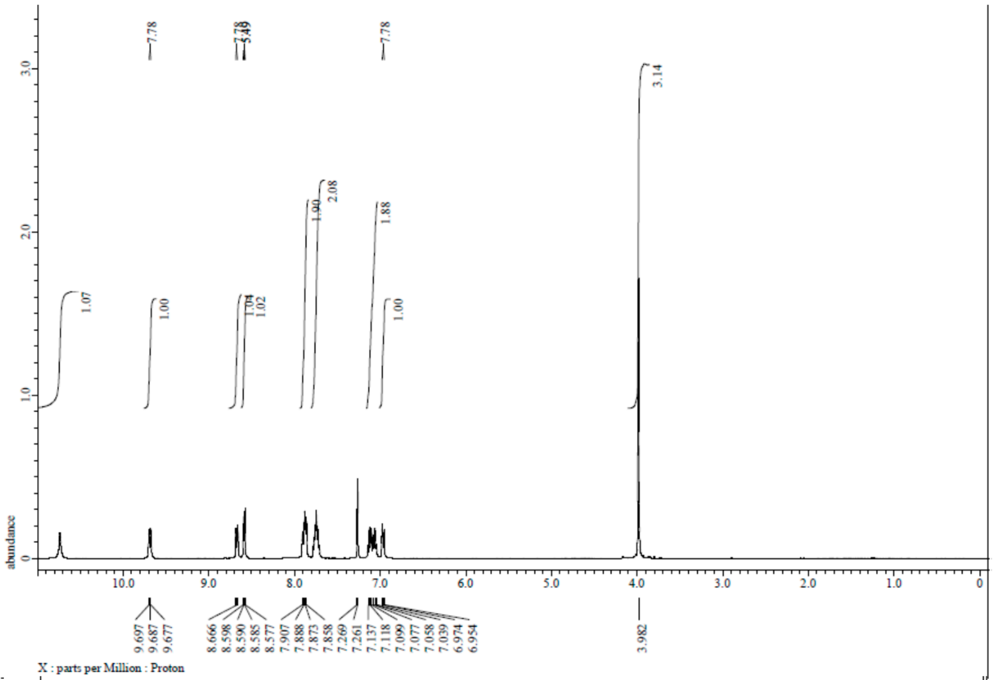

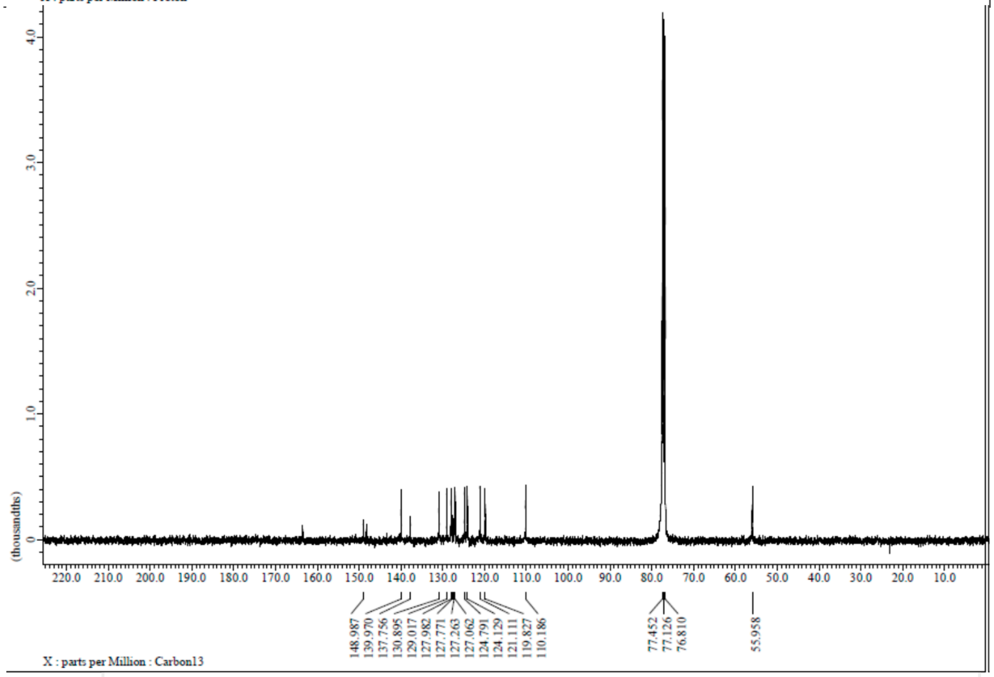

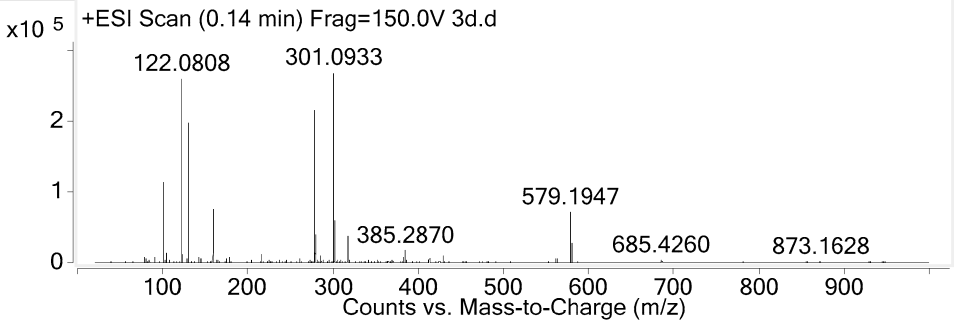

***N*-(3-Methoxyphenyl)isoquinoline-1-carboxamide (HSR1105)**

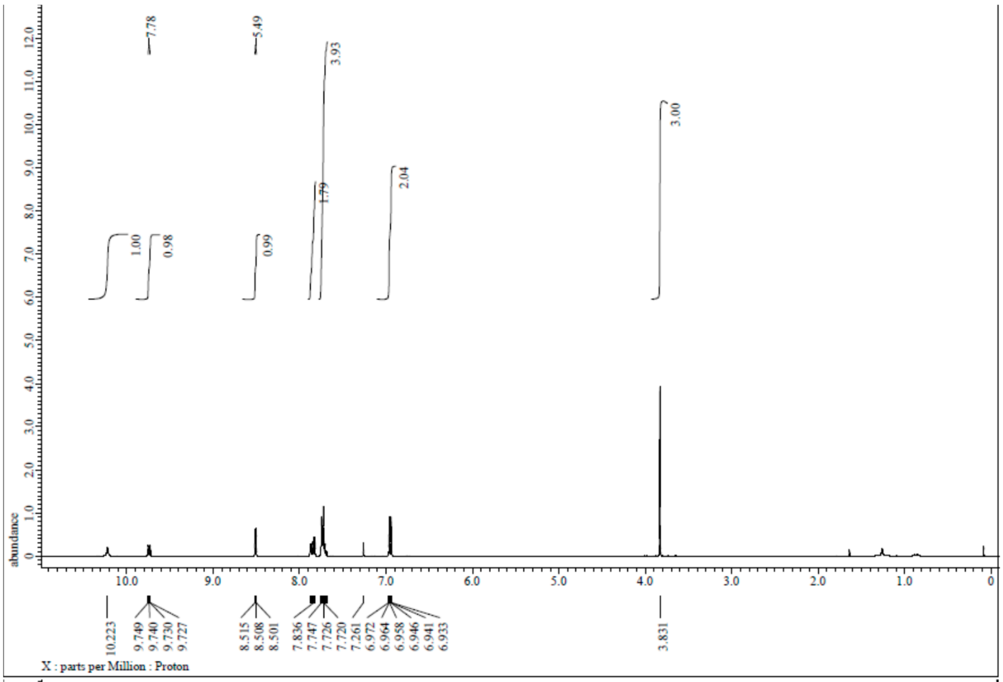

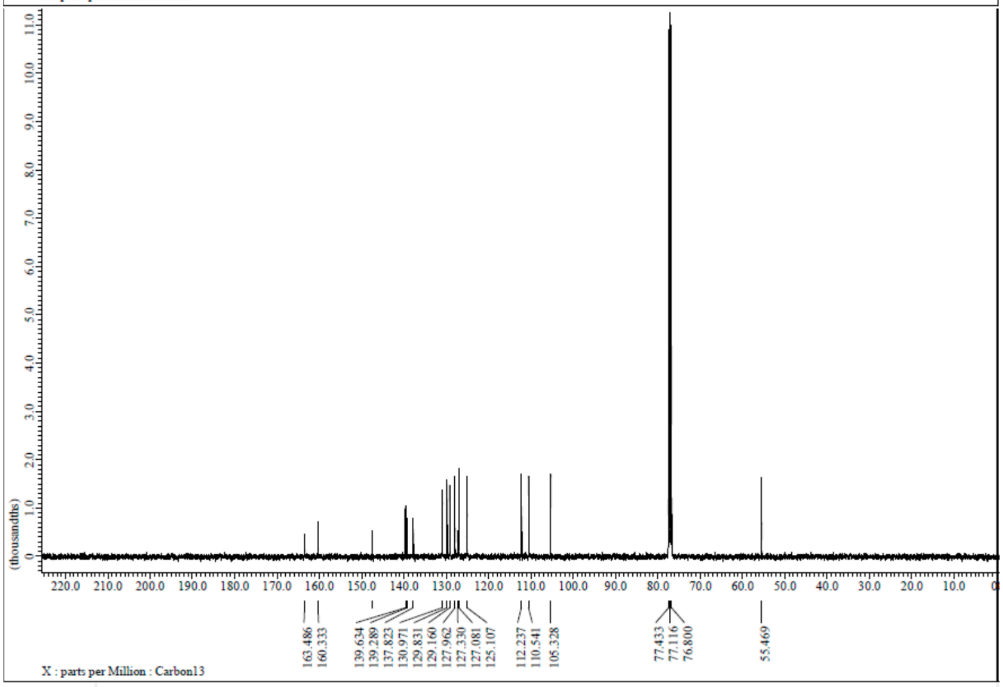

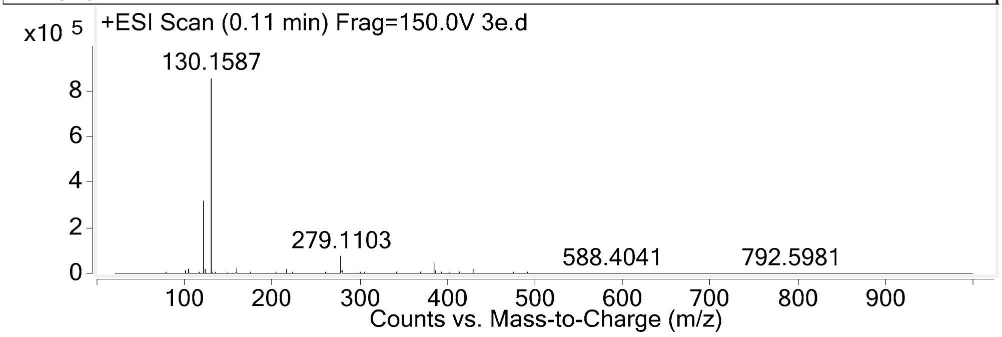

***N*-(4-Methoxyphenyl)isoquinoline-1-carboxamide (HSR1106)**

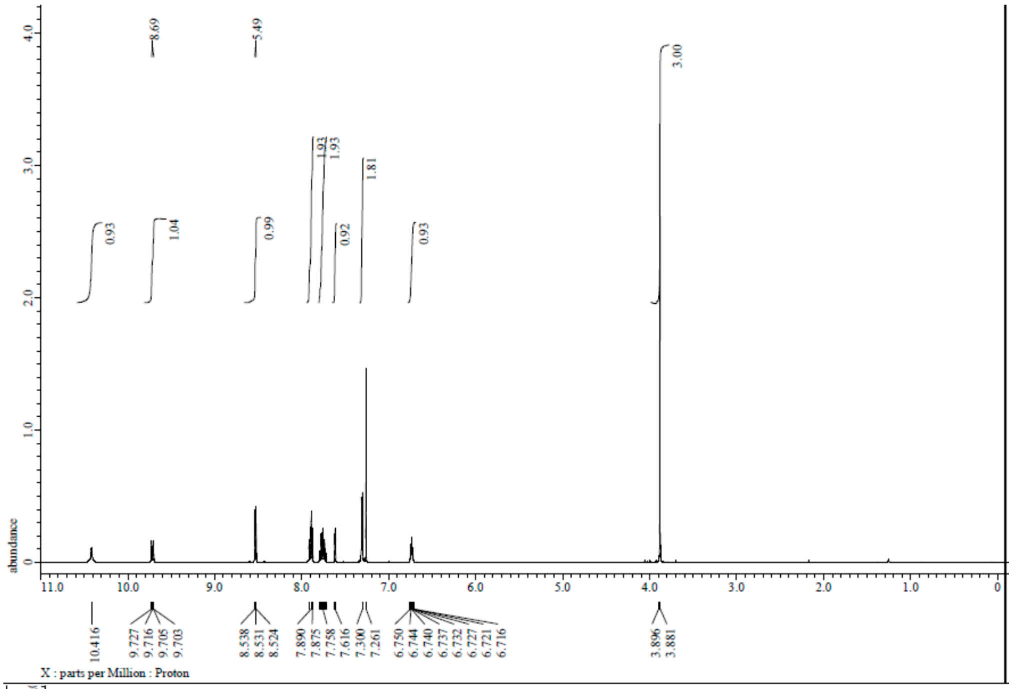

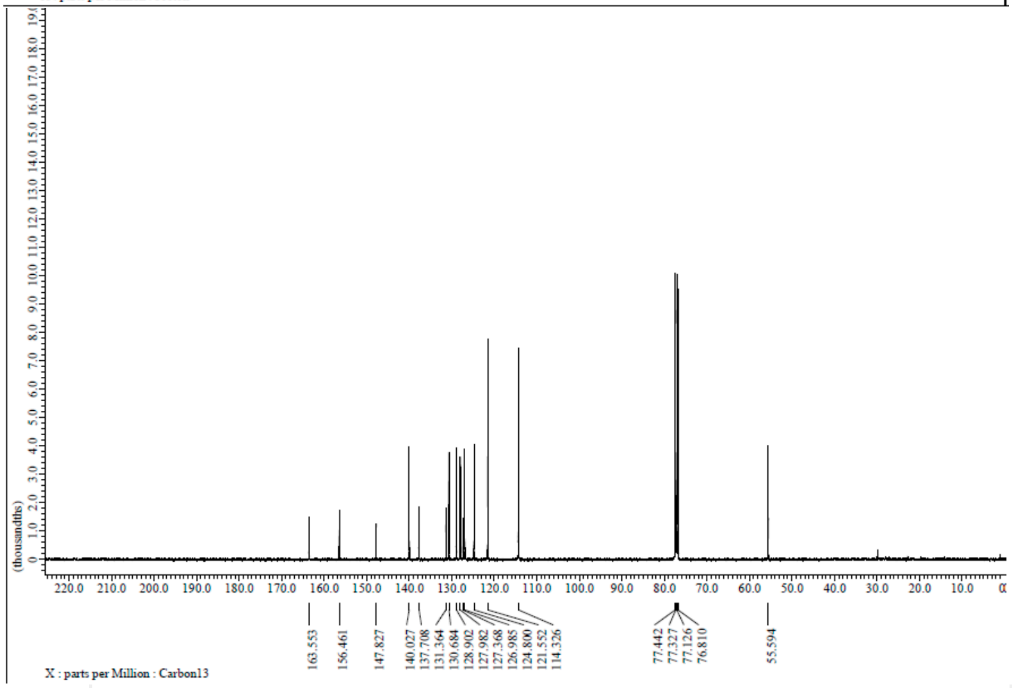

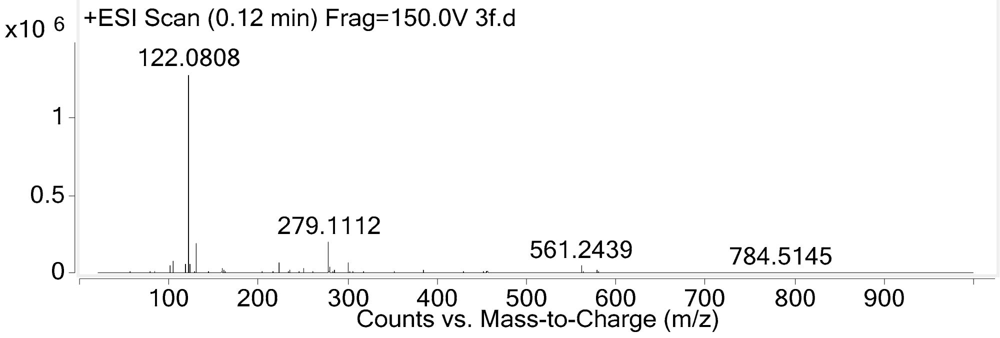

***N*-(2-(Trifluoromethyl)phenyl)isoquinoline-1-carboxamide (HSR1107)**

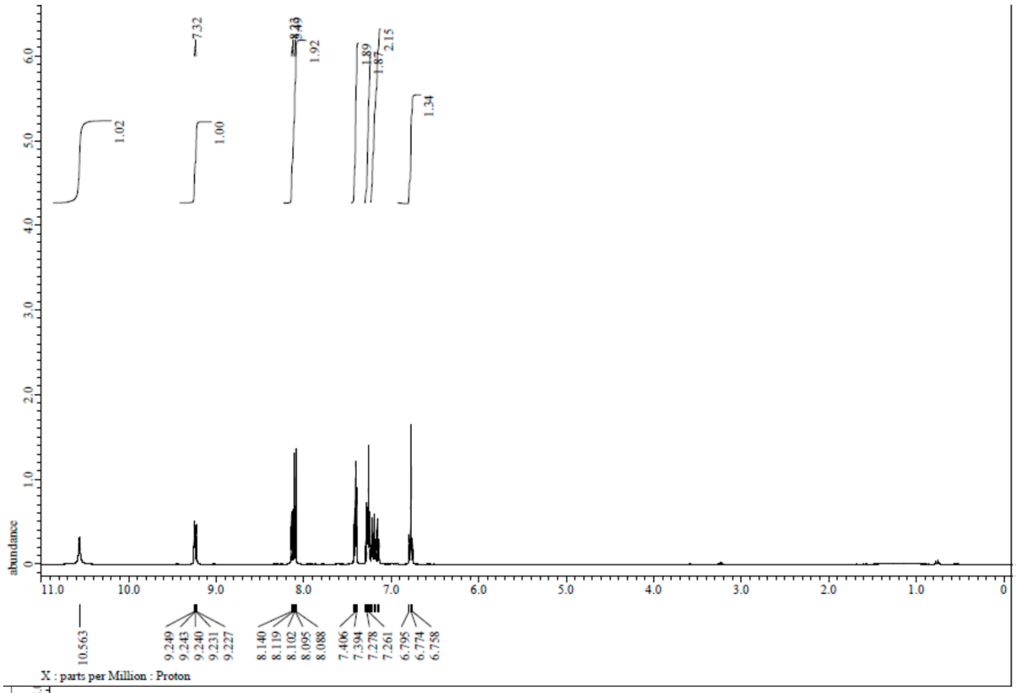

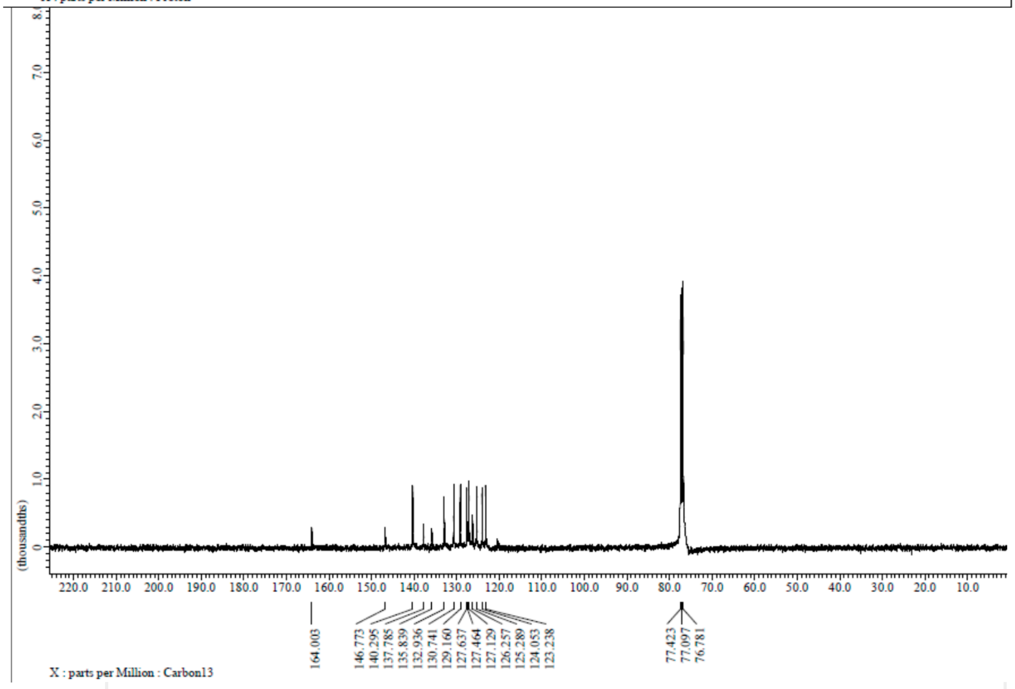

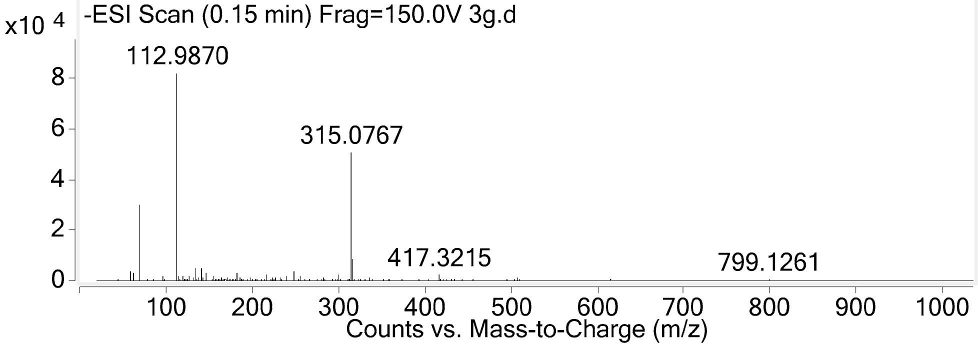

N-(3-(Trifluoromethyl)phenyl)isoquinoline-1-carboxamide (HSR1108)

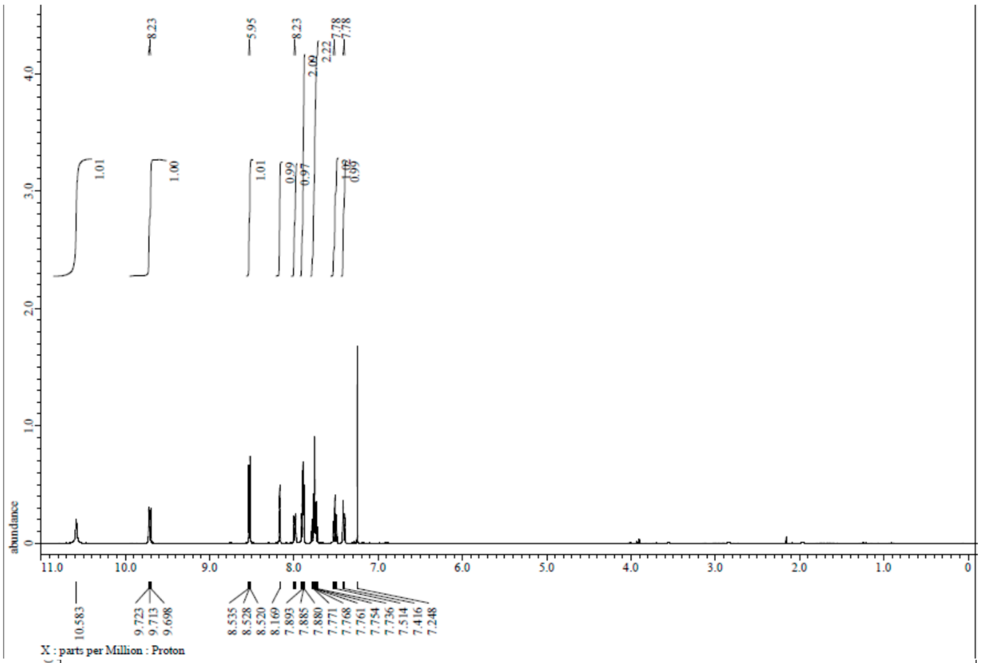

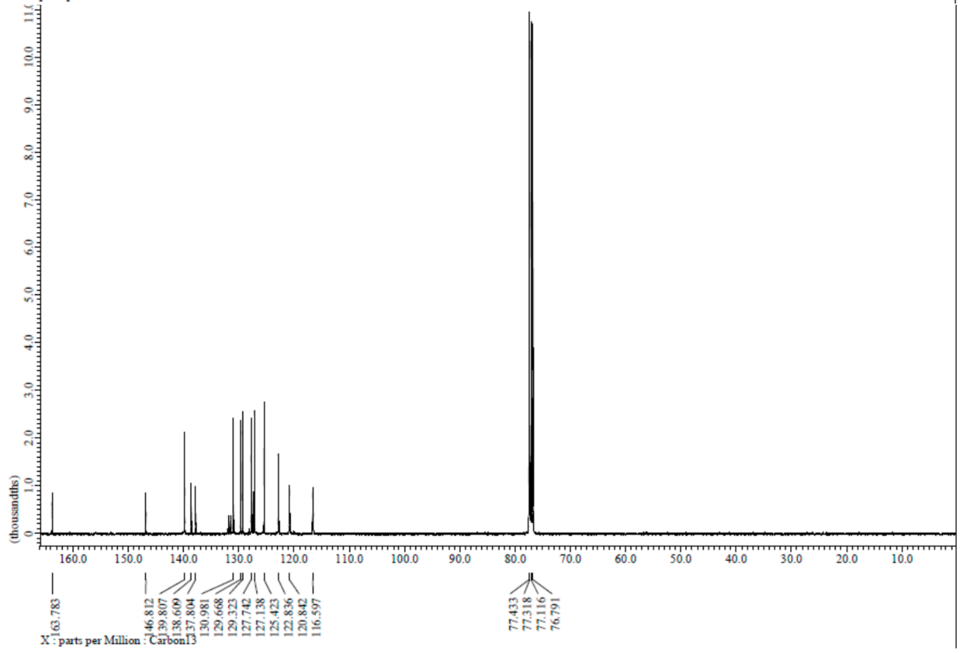

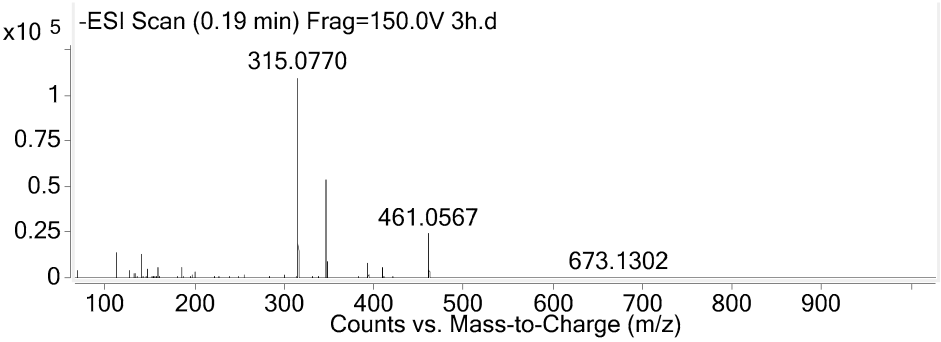

N-(4-(Trifluoromethyl)phenyl)isoquinoline-1-carboxamide (HSR1109)

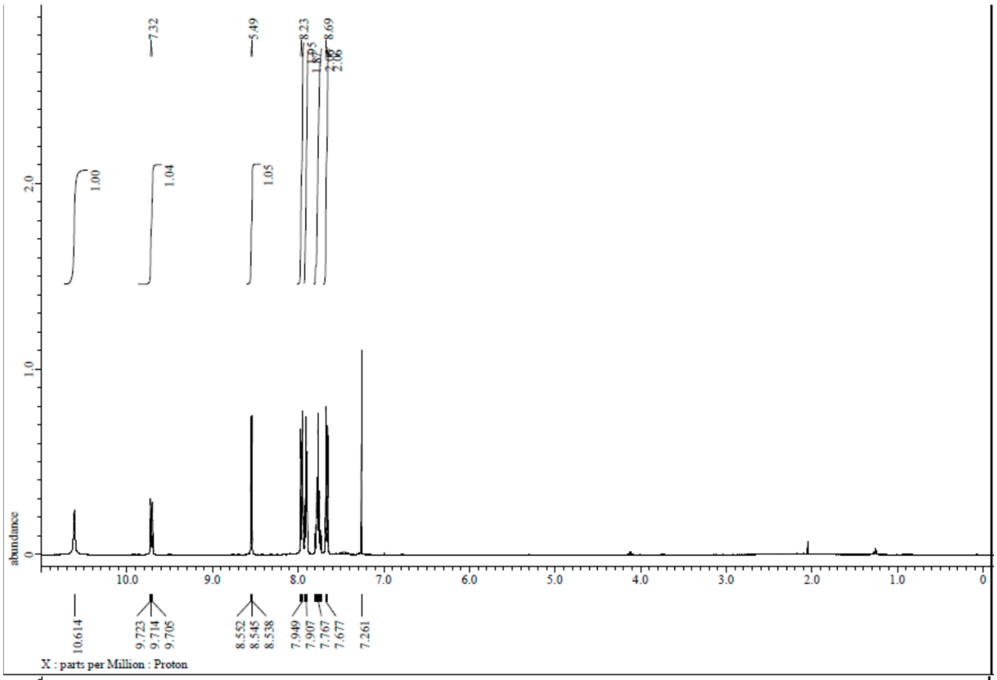

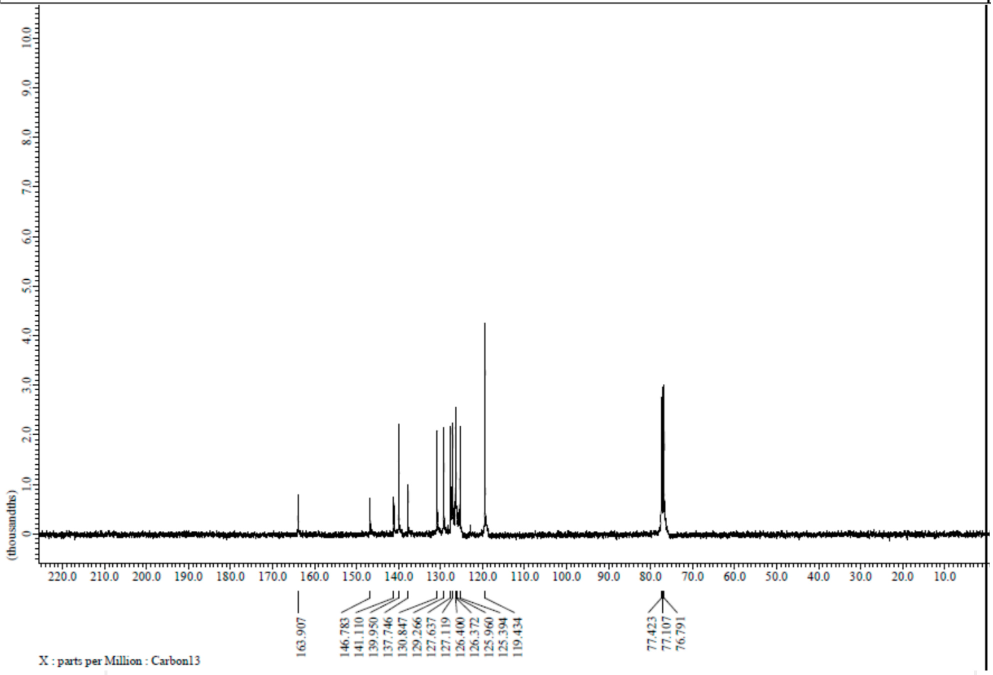

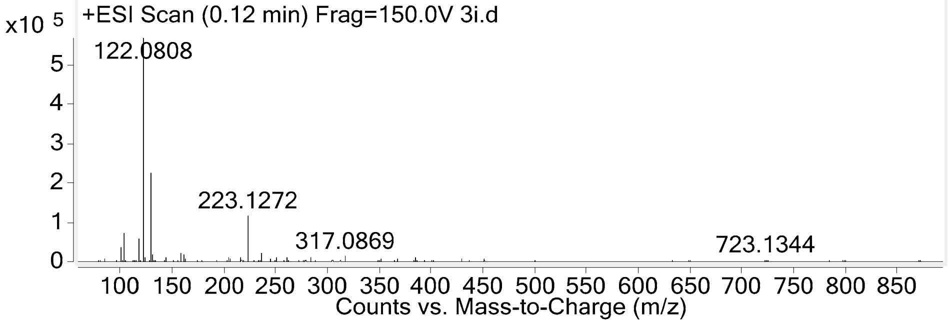

N-(3,5-Bis(trifluoromethyl)phenyl)isoquinoline-1-carboxamide (HSR1110)

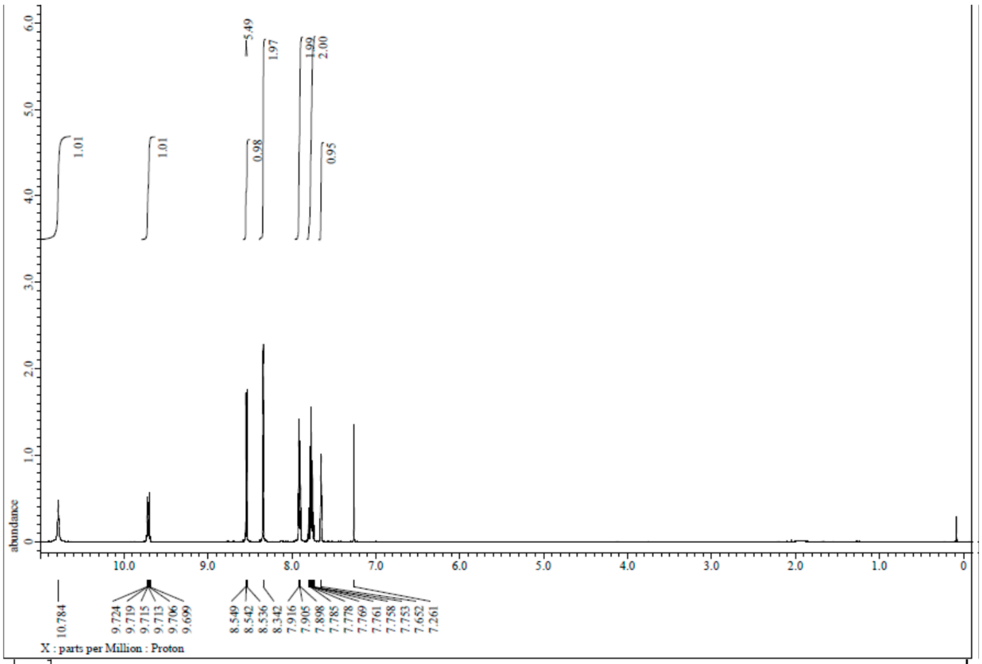

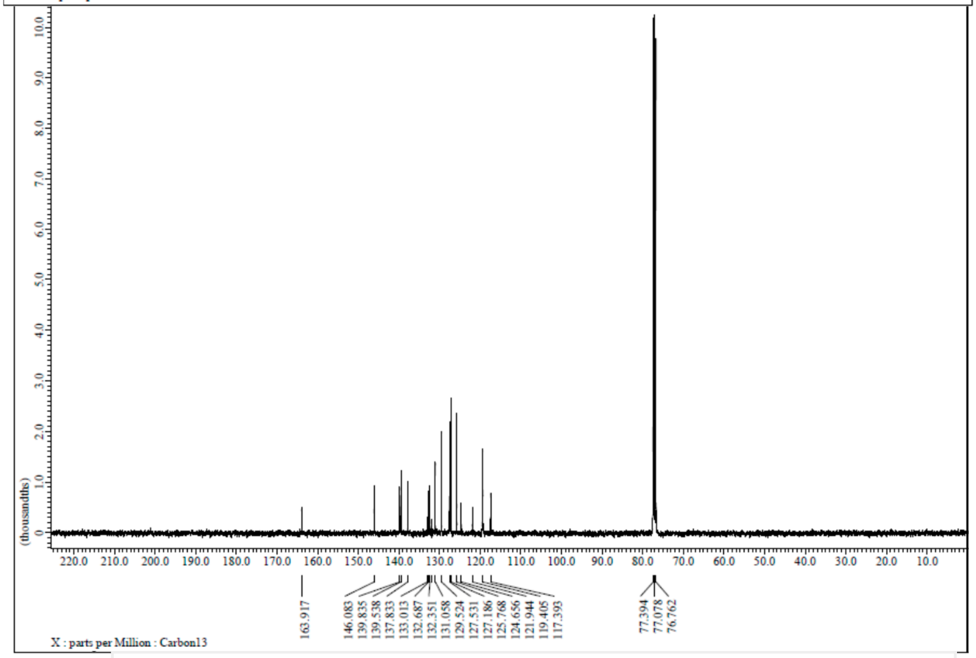

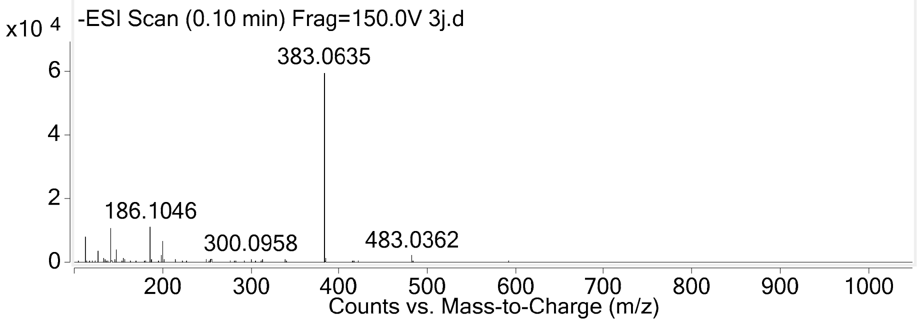

***N*-(3-Chlorophenyl)isoquinoline-1-carboxamide (HSR1111)**

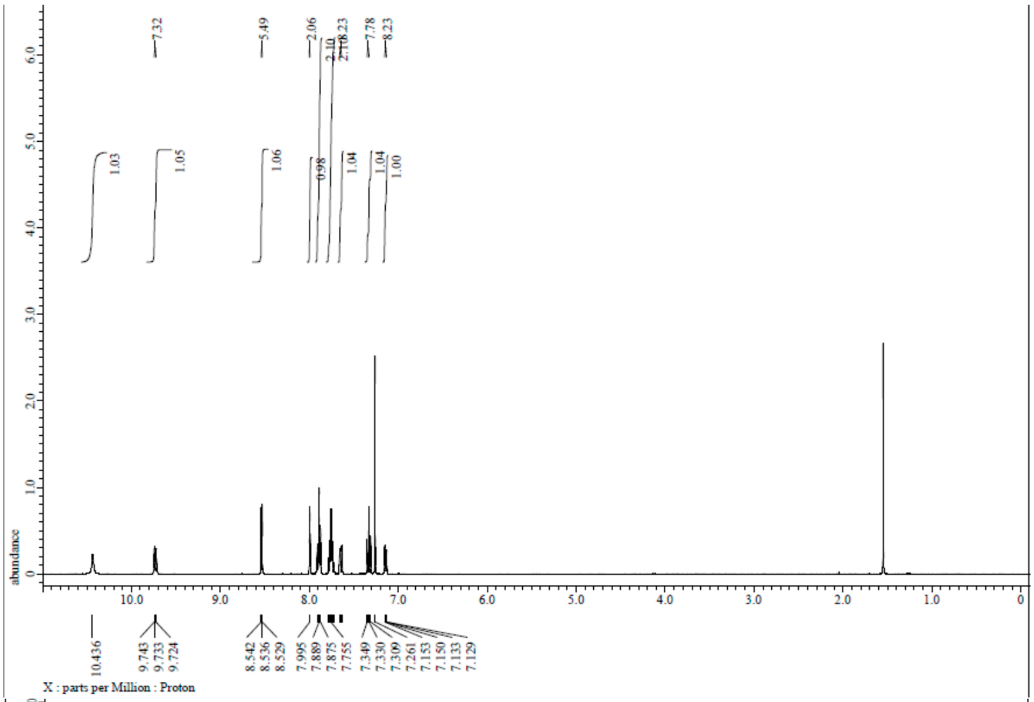

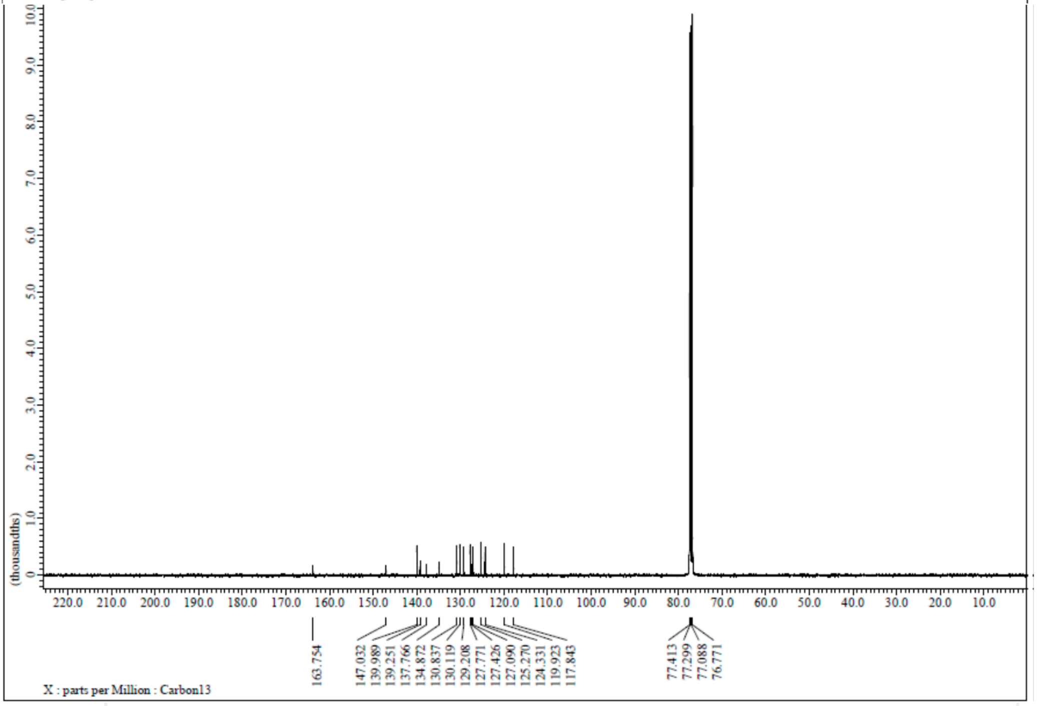

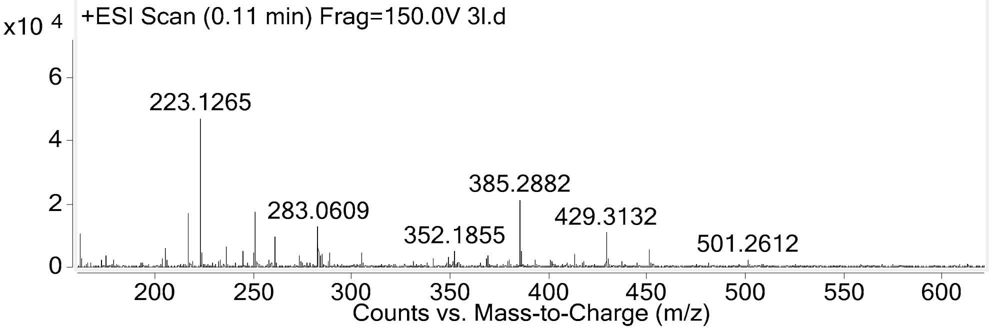

Supplement: Supplementary file 1 [file ijms-21-02319-s001.pdf]
